# USP3 promotes DNA damage response and chemotherapy resistance through stabilizing and deubiquitinating SMARCA5 in prostate cancer

**DOI:** 10.1038/s41419-024-07117-3

**Published:** 2024-11-05

**Authors:** Sheng Li, Situ Xiong, Zhongqi Li, Lin Yang, Hailang Yang, Jing Xiong, Wang Pan, Ju Guo, Songhui Xu, Bin Fu

**Affiliations:** 1 Department of Urology, The First Affiliated Hospital, Jiangxi Medical College, Nanchang University, Nanchang, Jiangxi China; 2 Jiangxi Provincial Key Laboratory of Urinary System Diseases, Nanchang, Jiangxi China; 3 College of Chemical and Environmental Engineering, Shanghai Institute of Technology, Shanghai, China

**Keywords:** Cancer, Cancer

## Abstract

The chromatin-remodeling enzyme SMARCA5 plays a key role in DNA-templated events including transcription, DNA replication, and DNA repair. Loss of function of the SMARCA5 can cause neurodevelopmental disorder and Williams syndrome. However, the molecular mechanism underlying the regulation of SMARCA5 in prostate cancer remains largely elusive. Here, we report that the deubiquitinating enzyme USP3 directly interacts with SMARCA5 and removes K63-linked polyubiquitination of SMARCA5 to maintain its stability, which promotes DNA damage repair and chemotherapy resistance. Depletion of USP3 or SMARCA5 promoted PCa cells sensitive to docetaxel and overexpression of USP3 restored the cells resistance to docetaxel treatment in SMARCA5 silenced cells in vitro and vivo. Clinically, USP3 was significantly up-regulated in prostate cancer tissues and positively associated with SMARCA5 expression. Collectively, our findings uncover a novel molecular mechanism for the USP3-SMARCA5 axis in regulating DSB repair with an important role in chemotherapy response in human prostate cancers, highlighting that targeting USP3-SMARCA5 axis could be a valuable strategy to treat USP3/SMARCA5-overexpressing chemotherapy-resistant patients and improve drug treatment.

## Introduction

Docetaxel-based chemotherapy is a standard-of-care treatment for metastatic prostate cancer, but only half of all patients will respond to docetaxel and the improvement in median survival of nearly two months with docetaxel in this elderly patient population, and will gradually develop to chemoresistance [1]. Recent studies have revealed that alterations in DNA damage response (DDR) genes are common in metastatic prostate cancer and can arise somatically in the tumor or can be inherited via the germline, which promoting chemotherapy resistance [2, 3]. Hence, factors in the DNA damage repair pathway serve as promising targets for overcoming chemotherapy resistance [4–6].

Protein ubiquitin modification is involved in almost all cellular processing, including cell cycle regulation, cell proliferation, cell death, differentiation, and metastasis; it acts by regulating protein stability, protein localization, and signal transduction [7]. The deubiquitinating enzymes (DUBs) function as removing ubiquitin molecules or polyubiquitin chains from substrates, which plays a crucial role in the DNA repair process and DNA damage response pathway [8–10]. Ubiquitin-specific protease 3 (USP3), a member of the USP family, has two domains: ZnF (1–158) and UCH (159–520) [11, 12]. Recent studies showed that USP3 is highly expressed in a variety of malignancies and is associated with a series of malignant biological behaviors of cancers [13–15], and also plays a key role in DNA damage response. For instance, after DNA damage, USP3 interacted with CHK1 and removed the K63-linked ubiquitin chain of CHK1, thus modulating the chromatin association and activation of CHK1 [16]. To further explore the potential roles of USP3 and its downstream mechanisms, we systematically detected the underlying proteins whose post-transcriptional modification might be involved in USP3-mediated deubiquitination by immunoaffinity purification and mass spectrometry (MS) analysis of USP3 interactors. Herein, we report one critical target of USP3, SMARCA5 and the specific mechanism of USP3-mediated deubiquitination of SMARCA5 in Docetaxel-inducing DNA damage, suggesting that USP3 may be a promising target for anticancer therapy in prostate cancer.

SMARCA5, the SWI/SNF-related matrix-associated actin-dependent regulator of chromatin subfamily A member 5, was required for DNA-templated events including transcription, DNA replication, and DNA repair [17, 18]. SMARCA5 is an ATPase from the ISWI subfamily that functions as a molecular motor for nuclear complexes that assemble and slide basic chromatin subunits, nucleosomes [18–20]. Currently, there is only a limited knowledge of how SMARCA5, which is highly expressed in cancers and the regulatory mechanism of SMARCA5 expression upregulation in cancers remains unknown. Here, we identified the posttranslational modification controlling SMARCA5 stabilization and the mechanism of SMARCA5 in regulating DNA damage response, which can be exploited for potential therapeutic interventions.

In the current study, we found that USP3 functions as the deubiquitinase of SMARCA5 and regulates the DDR. Mechanistically, USP3 binds SMARCA5 and removed K63-linked polyubiquitination of SMARCA5, which enhancing its stability. Knockdown of USP3 impairs the DDR through SMARCA5 and results in increased sensitivity to Docetaxel treatment in prostate cancer cells. In addition, we found that USP3 expression is up-regulated in prostate cancer tissues and positively with SMARCA5 expression. Moreover, USP3 knockdown sensitizes cancer cells to DNA-damaging agents in xenograft models, suggesting that the USP3- SMARCA5 axis may provide new therapeutic targets for overcoming chemotherapy resistance in prostate cancer.

## Materials and methods

### Cell lines and cell culture

HEK293T, PC3 and DU145 cells were purchased from ATCC and cultured in Dulbecco’s modified Eagle’s medium (DMEM) or RPMI1640 with supplemented with 10% FBS, 100 units per ml penicillin, and 0.1 mg per ml streptomycin. All the cell lines were routinely tested mycoplasma free by PCR and authenticated by short tandem repeat (STR) method and confirmed by National Infrastructure of Cell Line Resources of China.

### Histopathology

Histopathology was performed as previously [21]. In brief, prostate tissues, which were fixed in 10% neutral-buffered formalin overnight, were processed by standard procedures and embedded in paraffin. The paraffin-embedded tissues were sectioned (5 µm), deparaffinized, rehydrated and stained with hematoxylin and eosin (H&E), and Ki-67 by the clinical medical research center of the first affiliated hospital, Nanchang University. Histological analyses of the H&E- and Ki67-stained prostate tissues were performed by a board certified pathologist. Cell proliferation index was calculated as the percentage of Ki67-positive nuclei to the total number of nuclei.

### Immunohistochemistry (IHC)

Tissue sample collection was approved by the Internal Review and Ethics Boards of the First Affiliated Hospital of Nanchang University (registration number: (2023) CDYFYYLK(11-014)). Prostate tissue microarray were purchased from Aiotechnology (#SP159, http://www.dotarray.cn/) and the detailed information including stage, age, and classification and so on in the Supplementary Tables S1 and S3. Immunohistochemical (IHC) staining of USP3 (Proteintech, 18465-1-AP, dilution 1:100) and SMARCA5 (Santa Cruz Biotechnology, sc-365727, dilution 1:200) was carried out according to the standard protocol, as described previously [21]. The immunostaining was blindly scored by pathologists. The IHC score was judged as described in our previous publication. χ^2^ test and the Pearson correlation coefficient were used for statistical analysis of the correlation between USP3 and SMARCA5. The score of USP3 and SMARCA5 expression was classified semiquantitatively as follows: no staining: 0 point, weak staining: 1 point, moderate staining: 2 points, and strong staining: 3 points, whereas 2 and 3 were defined as high expression. The mean score from two pathologists was used as the final immunostaining score. The χ^2^ test was used for statistical analysis of USP3 and SMARCA5 expression.

### Mouse xenograft tumor assay

All BALB/c nude male mice (4–6 weeks of age) were obtained from Charles River Laboratories in China (Beijing). All animals used in this study received humane care in compliance with applicable regulations, policies, and guidelines relating to animals. All experimental procedures using animals were approved by the Institutional Animal Care and Use Committee of the first affiliated hospital of Nanchang University (registration number: CDYFY-IACUC-202304QR005). The indicated control PC3 cells (1 × 10^6^) and shUSP3#1 or shUSP3#2 were mixed with matrigel (1:1) and injected subcutaneously into the flanks of BALB/c nude male mice. For Fig. 5E–G Mouse xenograft tumor assay, the indicated control PC3 cells (1 × 10^6^), shUSP3 cells and shUSP3 combined overexpression of SMARCA5 were mixed with matrigel (1:1) and injected subcutaneously into the flanks of BALB/c nude male mice. For Fig. 6G–J Mouse xenograft tumor assay, the indicated PC3 cells stably expressing control shRNA vector (two groups), shUSP3 (two groups) were subcutaneously injected into the 6-week-old male BALB/C nude. When the mean size of tumor volume in each randomized group reached ∼100 mm^3^, the mice were randomly treated with docetaxel (20 mg/kg) every other day.

Tumors were measured using calipers every 7 days and tumor volumes were calculated using length × width × width × 0.5. Tumor tissues were paraffin embedded and H/E or Ki67 stained. Data were analyzed using analysis of variance (ANOVA) test.

### Statistical analysis

Statistical analyses were performed with Prism 8.0 (GraphPad Software). All statistical comparisons were evaluated by the Student’s *t* test or one-way or two-way analysis of variance (ANOVA). Among all the data sets, *p* values less than 0.05 were considered significant.

Additional materials and methods are presented in the Supplementary information.

## Results

### USP3 was frequently upregulated in PCa and correlates with prostate cancer progression

**Fig. 1:**
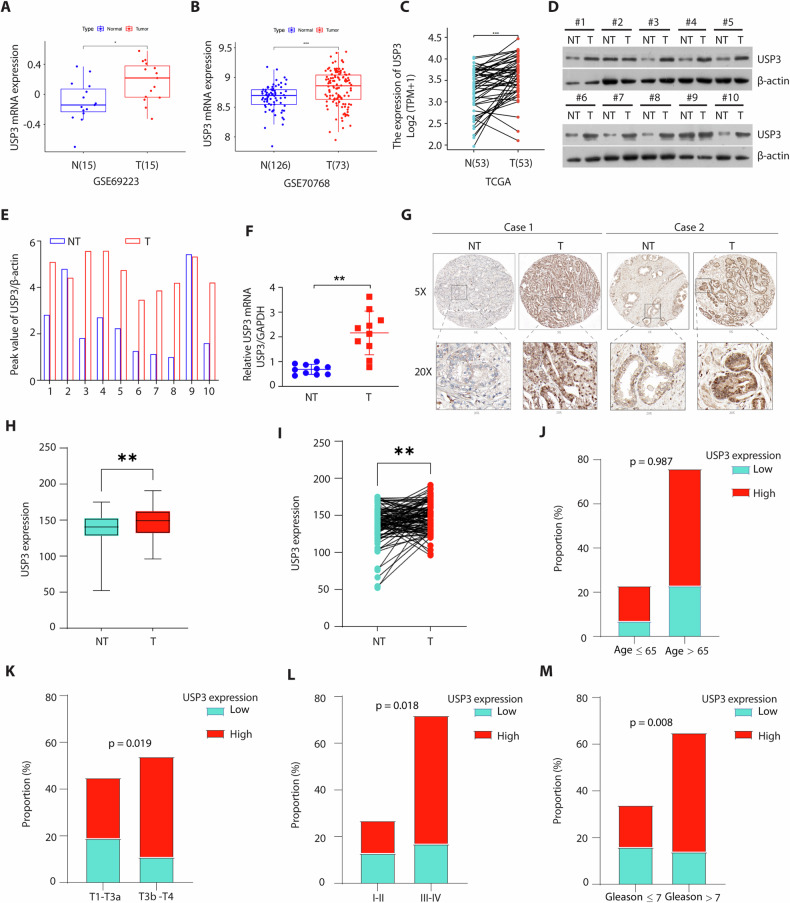
USP3 is elevated in PCa tissues and correlates with prostate cancer progression. **A**, **B** USP3 mRNA was overexpression in primary prostate cancer tissues compared with normal prostate tissues (GSE69223 and GSE70768). **C** Expression profile of USP3 mRNA in paired primary prostate cancer tissues (*n* = 53) and matched normal prostate tissues (*n* = 53). ^***^p < 0.001; TCGA. **D**, **E** USP3 protein expression in adjacent non-tumor tissues and matched PCa tissues was detected by western blot. USP3 protein expression was quantified using Image J software. **F** qPCR was used to detect the levels of USP3 mRNA in the above tissues. **G** Representative staining of USP3 in PCa tissues and adjacent non-tumor tissues. **H** USP3 protein expression was upregulated in adjacent non-tumor tissues (*n* = 99) and PCa tissues (*n* = 99). ^**^p < 0.01. **I** USP3 protein expression was upregulated in paired adjacent non-tumor tissues (*n* = 99) and matched PCa tissues (*n* = 99). ^**^p < 0.01. **J**–**M** A high USP3 expression was not related with age (*p* = 0.987), but related with a higher preoperative clinical T3b/T4 stage (*p* = 0.019), Tissue grade III-IV (*p* = 0.018) and Gleason score (*p* = 0.008). Relationship between USP3 expression and clinicopathological features. Chi-square tests were used to assess associations, with statistical significance indicated.

To dissect the contribution of USP3 to PCa, we first analyzed the data from GSE data base and TCGA. Surprisingly, we found that USP3 levels were upregulated in PCa compared with normal tissues (Fig. 1A–C) (Supplementary Fig. S1A). To validate this finding, we further assessed USP3 expression in our clinical PCa specimens. As shown in Fig. 1D–F, USP3 protein and mRNA expression was elevated in PCa tissues compared with adjacent non-tumor tissues. Next, we analyzed the expression of USP3 protein in tissue microarray containing 100 samples of PCa tissues and 99 samples of adjacent non-tumor tissues by Immunohistochemical (IHC) analysis (Supplementary Fig. S1B). IHC results were consistent with prior observations that USP3 is frequently upregulated in PCa (Fig. 1G–I). Of the 99 PCa tissue samples, 69 cases had a strong USP3 expression while only 30 cases had a weak or negative USP3 expression (Supplementary Table S1). Notably, a high USP3 expression was also correlated with larger tumor size and poor histological grade but not correlated with age and Lymph node metastasis (Fig. 1J–M) (Supplementary Table S1). These data indicate that elevated of USP3 may be involved in PCa progression.

### USP3 promotes proliferation and survival of prostate cancer cells

**Fig. 2:**
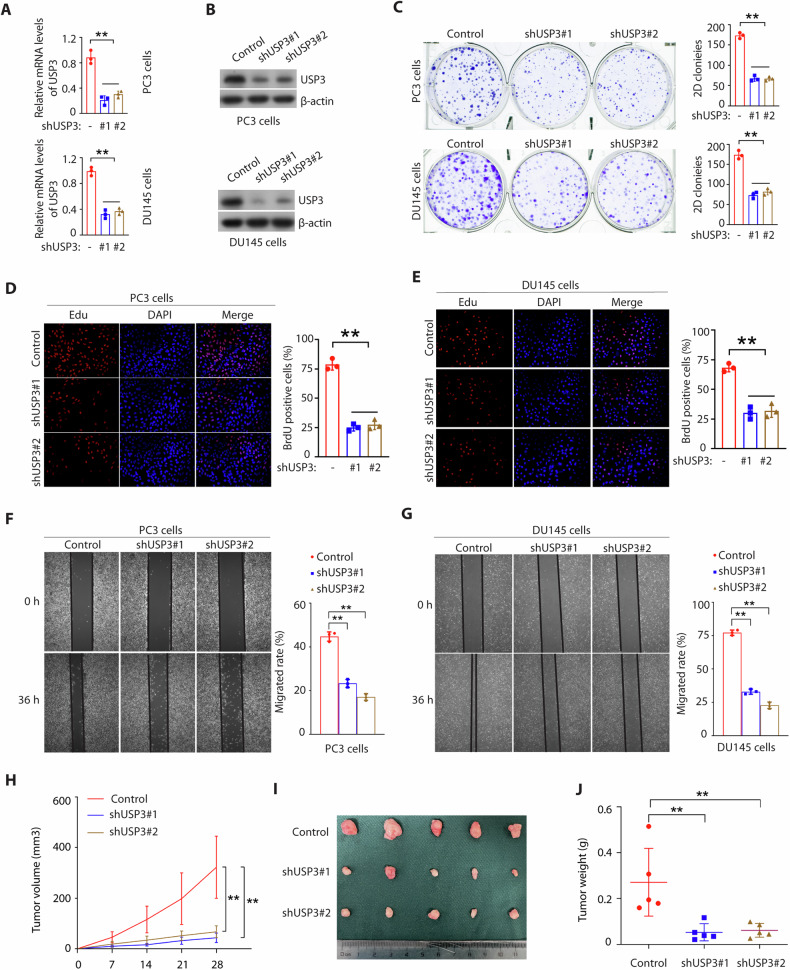
USP3 knockdown suppresses PCa cell proliferation in vitro and vivo. **A**, **B** USP3 was knocked down in PC3 and DU145 cells by lentivirus control plasmid or shUSP3 (#1 and #2), and were detected by qPCR and Western blot. ^**^p < 0.01. **C** The cells were generated as in **A** and colony-formation assays were performed (^**^p < 0.01). **D**, **E** Representative micrographs (left panel) and quantification (right panel) of Edu labeling in PC3 cells or DU145 cells stably expressing lentivirus control plasmid or shUSP3 (#1 and #2). ^**^p < 0.01. **F**, **G** The indicated cells as described in **A** were examined by wound healing assays. Representative micrographs (left panel) and quantification (right panel) of migrated cells. **H** PC3 shNC cells or shUSP3 cells were subcutaneously injected into Balb/c nude mice. Tumor volume growth curves from the indicated days and tumor growth was measured every 7 days. **I** After 28 days, mice were sacrificed and representative tumor images at the end of the experiment are presented. **J** Tumor weights were examined in the two groups. The statistical analyses were performed with the ANOVA. ^**^p < 0.01.

To further investigate USP3 function in prostate cancer, we knocked down USP3 in both PC3 and DU145 cells via lentiviral transduction using two different USP3 shRNAs. Separately, both constructs reduced expression of USP3 mRNA (Fig. 2A) and protein (Fig. 2B). USP3 knockdown reduced the proliferation of PC3 and DU145 cells determined by colony formation (Fig. 2C) and EdU assays (Fig. 2D, E), and also decreased the migration of both cells by wound-healing assay (Fig. 2F, G). In addition, USP3 knockdown also reduced proliferation in non-prostate cancer cells, suggesting that USP3 may be essential to proliferation in many cell lines (Supplementary Fig. S2A–D). To further test USP3 function in tumorigenesis, we employed an orthotopic prostate tumor model in which PC3 cells were injected subcutaneously into the nude mice. In control mice, injection of PC3 cells resulted in formation of large prostate tumors, while comparable injection of PC3 cells transduced with either one of two different USP3 shRNAs indicated that USP3 knockdown decreased tumorigenesis (Fig. 2H–J). Taken together, these data suggest that USP3 knockdown reduced long-term proliferation and survival of prostate cancer cells in vitro and in vivo.

### USP3 binds SMARCA5 and removed K63-linked polyubiquitination of SMARCA5

**Fig. 3:**
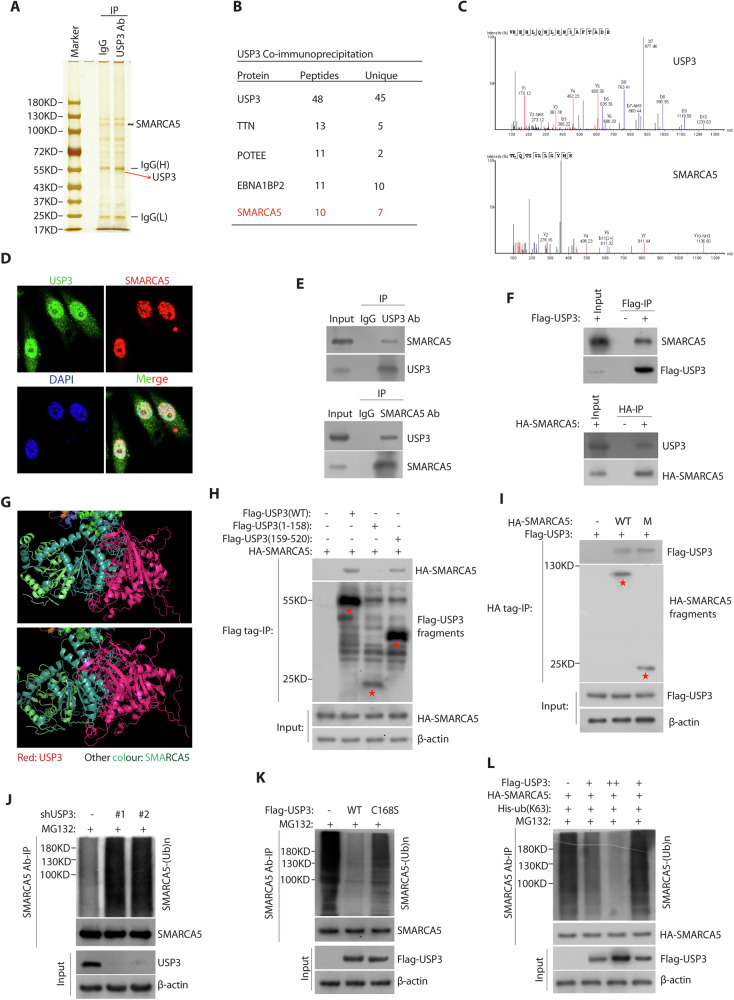
USP3 directly interacts with and deubiquitinates SMARCA5. **A** The PC3 cells were lysed and subjected to immunoprecipitation using anti-USP3 antibody and followed by protein A/G agarose beads. The complexes were then separated, and the gels were stained with silver. **B** List of USP3-associated proteins identified by mass spectrometric analysis. **C** Representative best unique peptides of USP3 and SMARCA5 were identified by mass spectrometry assays. **D** Immunostaining of USP3 (green) and SMARCA5 (red) were detected by their antibody in PC3 cells. Nuclear 4’, 6-diamidino-2-phenylindole (DAPI; blue). **E**, **F** The interaction between USP3 and SMARCA5 in PC3 cells was detected by co-immunoprecipitation assay. **G** The both binding interface between USP3 and SMARCA5 was based on the molecular docking model. **H** HEK293T cells were cotransfected with HA-SMARCA5 and Flag-tagged full-length USP3 or its deletion mutants, and cell lysates were subjected to IP and detected with the indicated antibodies. **I** HEK293T cells were co-transfected with Flag-USP3 and HA-tagged full-length SMARCA5 or its deletion mutant (487–638) and cell lysates were subjected to IP and detected with the indicated antibodies. **J** The PC3 cells stably expressing control or USP3 shRNA#1 and or #2 were subjected to deubiquitination assay and the polyubiquitylated SMARCA5 protein was detected by the anti-Ub antibody. **K** The PC3 cells were transfected with Flag-USP3 and Flag-USP3 (C168S) as indicated. The polyubiquitylated SMARCA5 protein was detected by the anti-Ub antibody. **L** HEK293T cells were transfected with Flag-USP3 (+means 2 μg, ++ means 4 μg), HA-SMARCA5, and His-ub (K63). The polyubiquitylated SMARCA5 protein was detected by the anti-Ub antibody.

To elucidate the molecular mechanism of USP3-mediated PCa progression, we performed immunoaffinity purification and mass spectrometry (MS) analysis of USP3 interactors (Fig. 3A). Mass spectrometry analysis (Fig. 3B, C) and the gene ontology analysis of USP3 interactome (Supplementary Fig. S3A, B) showed that USP3 interacted proteins mainly enriched on nuclear division. Among the TOP4 interacted proteins (TTN, POTEE, EBNA1BP2 and SMARCA5) (Supplementary Tables S2), only SMARCA5, a chromatin-remodeling enzyme, was overexpression in PCa tissues (Supplementary Fig. S3C) and required for DNA-templated events including transcription, DNA replication, and DNA repair [18]. On the other hand, previous studies showed that USP3 play a key role in DNA repair [11, 16, 22]. So we asked whether USP3 interacted with SMARCA5 to play function in prostate cancer. Our immunofluorescence staining revealed that endogenous USP3 and SMARCA5 were mainly colocalized in the nuclear in both two PCa cells (Fig. 3D) (Supplementary Fig. S3D) and then we found that endogenous USP3 and SMARCA5 coprecipitated in PC3 cells (Fig. 3E). We further confirm that Flag-tagged USP3 or HA-tagged SMARCA5 could interact with endogenous SMARCA5 or USP3 in PC3 cells (Fig. 3F). Based on binding models of USP3 with SMARCA5 (Fig. 3G), the amino residues (Ser286/Ser286/Asn304/Ser284/Pro336/Asn252/Tyr267 were located in the UCH domain of USP3) of USP3 was forming hydrogen bonds with the matched amino residues (Lys600/Gly499/Lys496/Asn549/Arg522/Glu516/Tyr526 were located in the Helicase C-terminal of SMARCA5) of SMARCA5. The USP3 protein has been known to have two domains: ZnF (1–158) and UCH (159–520) [11, 12]. Here, we found that the UCH domain of USP3 could interact with the Helicase C-terminal of SMARCA5 (Fig. 3H, I), which consistent with the binding models. Then, we hypothesized that USP3, a deubiquitinase ligase, regulated SMARCA5 through deubiquitination. Firstly, we found USP3 silencing increased SMARCA5 polyubiquitination (Fig. 3J). Second, ectopic expression of wild-type USP3, but not the C168S mutant, reduced the polyubiquitination of SMARCA5 (Fig. 3K), suggesting that the enzymatic activity of USP3 is indispensable for USP3-dependent deubiquitination of SMARCA5. Furthermore, we revealed that USP3 promotes K63-linked deubiquitination of SMARCA5 (Fig. 3L) (Supplementary Fig. S3E). USP3 removes the deubiquitination of SMARCA5, so which protein mediates the ubiquitination process of SMARCA5? Previous studies report that E3 ubiquitin ligase RNF168 and RNF180 regulated ubiquitination of SMARCA5 in response to DNA damage [23–25]. Here, we found that RNF168 knockdown reduced ubiquitination of SMARCA5, but RNF180 knockdown has no effect, suggesting that RNF168 may mediate the ubiquitination process of SMARCA5 (Supplementary Fig. S3F, G). This needs further investigation in the next project. Taken together, these findings demonstrate that USP3 interacted with SMARCA5 and removed K63-linked polyubiquitination of SMARCA5.

### USP3 regulated SMARCA5 stability and positively correlates with SMARCA5 in PCa specimens

**Fig. 4:**
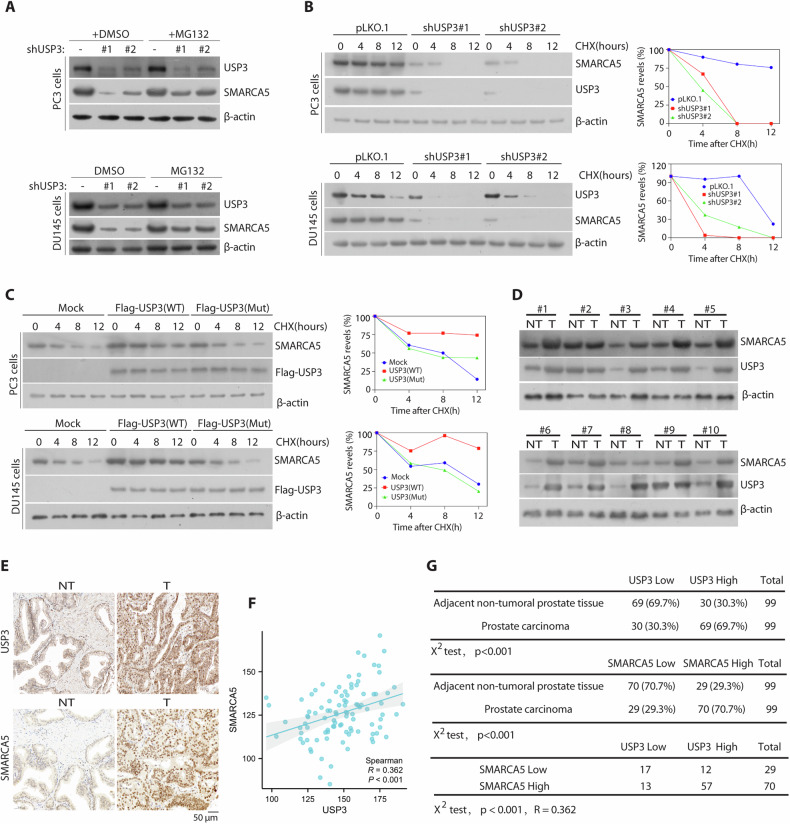
USP3 is a bona fide DUB targeting SMARCA5 protein for deubiquitination and stabilization. **A** The PCa cells transfected with the indicated constructs were treated with or without MG132 for 4 h before harvest. USP3 and SMARCA5 protein were detected by the indicated antibodies. **B** PC3 cells (up) and DU145 cells (down) were transduced with USP3 shRNA#1 and or #2, treated with 50 mg/mL cycloheximide, harvested at different time points, and then immunoblotted with antibodies to USP3, SMARCA5 and β-actin. Right, quantification of SMARCA5 protein levels (normalized to β-actin). **C** The cells were transfected with Flag-USP3 (WT) or Flag-USP3 (Mut) for 48 h, then analyzed and quantified as described as in **B**. **D** USP3 protein were upregulated in 10 freshly collected paired human prostate tumor tissues (T) and matched adjacent non-tumor tissues (NT), and positively related with SMARCA5 expression. **E** Representative staining of USP3 and SMARCA5 in paired adjacent non-tumor tissues (*n* = 99) and matched PCa tissues. **F** The USP3 protein levels were positively correlated with SMARCA5 protein levels in human prostate tumor tissues. **G** USP3 and SMARCA5 protein expression status in adjacent nontumoral prostate tissue and prostate carcinoma specimens, and the correlation study of USP3 and SMARCA5 expression level in PCa tissues. Statistical analyses were undertaken with the χ^2^ test, *P* < 0.001. R, Pearson’s correlation coefficient.

To address whether and how USP3 stabilized SMARCA5, we treated the indicated PCa cells with or without proteasome inhibitor MG132 and examined the protein levels (Fig. 4A). We found that the treating cells with the proteasome inhibitor MG132 significantly increased the decreased SMARCA5 protein level in cells silenced of USP3 (Fig. 4A). To further establish that USP3 regulates SMARCA5 stability, we treated cells with cycloheximide (CHX) and determined the half-life of SMARCA5. As shown in Fig. 4B, USP3 knockdown promoted the destabilization of the SMARCA5 protein in both PCa cells. Conversely, overexpression of USP3, but not the C168S mutant, led to a prominent increase in the stability of endogenous protein, whereas the stability of USP3 was not affected (Fig. 4C). Moreover, USP3 knockdown did not affect the mRNA level of SMARCA in both PC3 and DU145 cells (Supplementary Fig. S4A, B). Taken together, these results suggest that USP3 targets SMARCA5 for stability.

As USP3 and SMARCA5 were required for DNA repair [18, 22], which has a key role in human cancer, it is possible that USP3 promotes deubiquitination and stabilization of SMARCA5 in PCa specimens. We then detected the expression of USP3 and SMARCA5 in PCa tissue samples. We found the high USP3 protein levels were correlated with increased SMARCA5 in most of the PCa tissues (Fig. 4D). To further confirm the results, we performed immunohistochemical staining of USP3 and SMARCA5 in PCa samples. We also observed that higher expression of USP3 was positively associated with stronger expression of SMARCA5 in tumor tissues (Fig. 4E). Pearson’s correlation analyses revealed a significant positive correlation between the expression scores of USP3 and SMARCA5 (R = 0.362, *P* < 0.001) (Fig. 4F). Of the 99 PCa tissue samples, 70 cases had strong SMARCA5 expression and among them there are 57 cases having a strong USP3 expression (Fig. 4G). A high SMARCA5 expression was also correlated with larger tumor size and poor histological grade but not correlated with age and Lymph node metastasis (Supplementary Table S3). Taken together, these results indicate that USP3 is upregulated in prostate cancer, positively correlating with SMARCA5 expression.

### USP3 regulates DNA damage response through SMARCA5

**Fig. 5:**
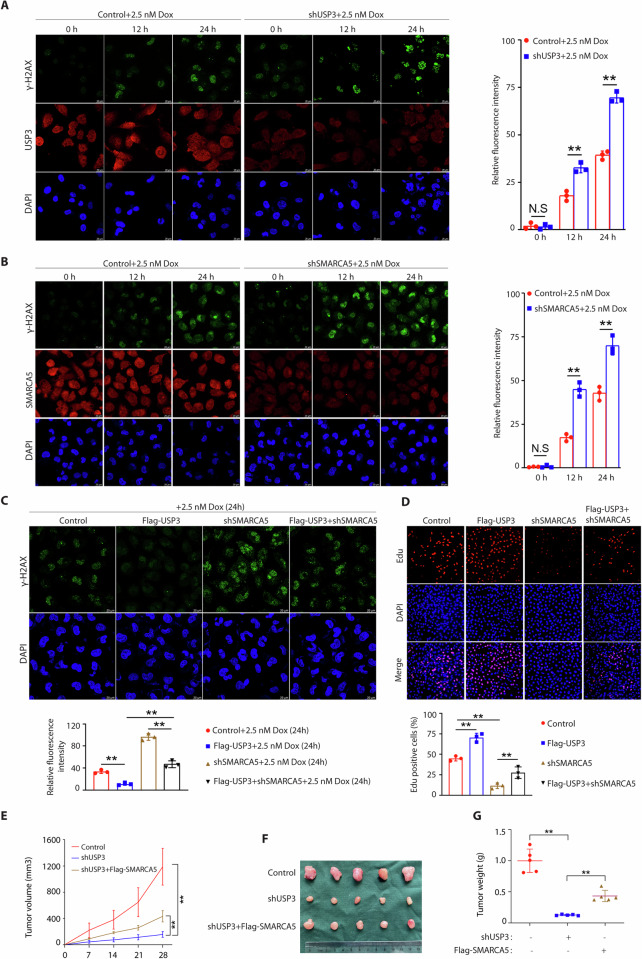
USP3 regulates DNA damage response (DDR) through SMARCA5. **A** The PC3 cells with or without USP3 silencing were treated with 2.5 nM docetaxel (Dox) at the indicated time points before fixing and processed for γ-H2AX immunofluorescence. N.S means no significance. ^**^p < 0.01. **B** The PC3 cells with or without SMARCA5 silencing were treated with 2.5 nM docetaxel (Dox) at the indicated time points before fixing and processed for γ-H2AX immunofluorescence. N.S means no significance. ^**^p < 0.01. **C** Flag-USP3 rescued SMARCA5 knockdown-mediated high levels of γ-H2AX post-docetaxel treatment at the indicated time points. Cells as described in Supplementary Fig. S5G were fixing and processed for γ-H2AX immunofluorescence. **D** Representative micrographs (left panel) and quantification (right panel) of Edu labeling in PC3 cells stably expressing lentivirus control plasmids or other indicated plasmids (Flag-USP3, shSMARCA5 or Flag-USP3+shSMARCA5). ^**^p < 0.01. **E**–**G** The indicated PC3 cells (Control, shUSP3 or shUSP3+Flag-SMARCA5) were subcutaneously injected into Balb/c nude mice. Tumor volume growth curves from the indicated days and tumor growth was measured every 7 days (**E**). **F** After 28 days, mice were sacrificed and representative tumor images at the end of the experiment are presented. **G** Tumor weights were examined in the two groups. The statistical analyses were performed with the ANOVA. ^**^p < 0.01.

Loss of SMARCA5 impaired DNA end resection and increased DNA damage that can be detected by higher levels of γ-H2AX staining [18, 26, 27]. To explore the physiologic function of USP3- SMARCA5 axis in the DNA damage process, we detected γ-H2AX foci formation in USP3 or SMARCA5 silenced cells after exposure to 2.5 nM docetaxel (Supplementary Fig. S3A, B showed 2.5 nM docetaxel could induce obvious morphological changes of two PCa cells). As shown in Fig. 5A, B and Supplementary Fig. S5C–F, these results revealed significantly higher γ-H2AX induction in USP3 or SMARCA5 silenced cells after docetaxel treatment in a time dependent manner. Moreover, we found overexpression of USP3 could decrease γ-H2AX induction and partially rescued SMARCA5 knockdown-mediated high levels of γ-H2AX post-docetaxel treatment at the indicated time points in both PC3 and DU145 cells (Supplementary Fig. S5G–I, Fig. 5C). Besides, we demonstrated that SMARCA5 knockdown decreased cell proliferation and significantly rescued the effect of overexpression of USP3 (Fig. 5D). To test the biological function of USP3-SMARCA5 axis in in vivo, we employed an orthotopic prostate tumor model in which PC3 cells were injected subcutaneously into the nude mice. USP3 knockdown inhibited tumor growth, while co-overexpression of SMARCA5 significantly rescued this effect (Fig. 5E–G). Overall, these data suggest that USP3 regulates DNA damage response in a SMARCA5 dependent manner.

### USP3 promotes chemotherapy resistance via SMARCA5

**Fig. 6:**
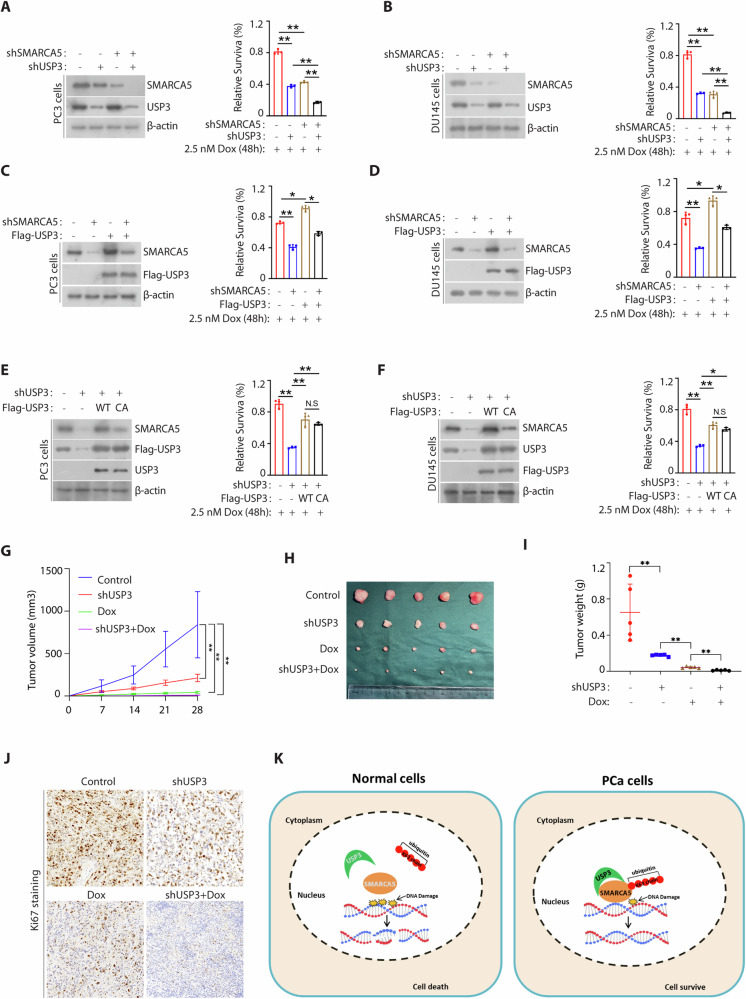
USP3 regulates chemotherapy resistance via SMARCA5. **A**, **B** The PC3 cells and DU145 cells stably expressing USP3 shRNA were transfected with or without SMARCA5 shRNA and cells were analyzed by Western blotting for indicated proteins. Surviving cell percentage was counted after treated 2.5 nM docetaxel with for 2 days (mean ± SD, *n* = 3). **C**, **D** The PC3 cells and DU145 cells stably expressing Flag-USP3 were transfected with or without SMARCA5 shRNA and cells were analyzed by Western blotting for indicated proteins. Surviving cell percentage was counted after treated 2.5 nM docetaxel for 2 days. **E**, **F** The PC3 cells and DU145 cells stably expressing USP3 shRNA were transfected with Flag-USP3 (WT) or Flag-USP3 (CA) and cells were analyzed by Western blotting for indicated proteins. Surviving cell percentage was counted after treated 2.5 nM docetaxel for 2 days. **G**–**J** Tumor xenograft assays were performed by subcutaneous injection of PC3 cells stably expressing USP3 shRNA or control. Tumor growth rate in nude mice treated every other day with docetaxel (20 mg/kg) is shown **G**. Tumors were dissected and recorded after euthanizing the mice (**H**). Mice were sacrificed after 28 days. Tumor weights were measured as shown in **I**. Representative data are shown from five mice each group by two-sided unpaired t-test. **J** Representative staining of Ki67 on the tumor sections derived from above treatment mice. The staining was developed by DAB (brown) and counterstained by hematoxylin (blue). Statistical analyses were performed with the ANOVA, ^*^p < 0.05; ^**^p < 0.01. **K** Schematic model showing that USP3 deubiquitinates and stabilizes SMARCA5 by K63-linked deubiquitin, which promotes DNA damage response.

As well known that the status of the DNA damage response pathway has an effect on cancer cell response to chemotherapy, and loss of DNA damage response elements induces sensitivity to DNA-damaging agents [28, 29]. To test whether the potential role of the USP3-SMARCA5 axis may as a target for cancer therapy, we silenced USP3 or SMARCA5 individually or together in PC3 cells or DU145 cells (Fig. 6A, B: left), and examined the cell survival following docetaxel. We revealed that USP3 or SMARCA5 silencing promoted PC3 cells and DU145 cells sensitive to docetaxel (Fig. 6A, B: right). Moreover, silencing of SMARCA5 in USP3 depleted cells did further sensitize cells to these treatments (Fig. 6A, B: right). Similar results were observed in the treatment with etoposide or zeocin (Supplementary Fig. S6A–D). Overexpression of USP3 promoted cells resistant to docetaxel treatment and SMARCA5 silencing in both PCa cells resensitized cells to docetaxel in USP3 overexpressed cells (Fig. 6C, D). To further confirm that the regulation of resistance to chemotherapy by USP3 is dependent on its catalytic activity, we reconstituted USP3 silenced cells with overexpression of wild type USP3 or C168 mutant plasmid (Fig. 6E, F) and detected the cell survival upon docetaxel. As shown in Fig. 6E, F, USP3 silencing sensitized cells to docetaxel. However, reconstitution of wild type USP3 but not the C168 mutant restored these phenotypes, suggesting that the catalytic activity of USP3 is important for its regulation of the cellular response to docetaxel treatment. Next, we further asked whether USP3 may as a potential target for prostate cancer therapy in vivo. As shown in Fig. 6G–I, depletion of USP3 or docetaxel treatment similarly inhibited tumor growth, whereas the combined silencing of USP3 and docetaxel treatment did further reduce tumor growth in the xenograft model. Similar effects were observed in Ki67 staining assays (Fig. 6J). We also confirmed that USP3 were indeed knocked down and USP3 knockdown decreased the expression of SMARCA5 while docetaxel treatment has no effect on their protein expression in respective tumor tissues (Supplementary Fig. S6E). These data demonstrate that USP3 regulates cellular response to docetaxel in prostate cancer cells in a SMARCA5 dependent manner.

Collectively, we reveal that USP3 binds SMARCA5 and removed K63-linked polyubiquitination of SMARCA5 to maintain its stability, which promotes DNA damage repair and chemotherapy resistance (Fig. 6K).

## Discussion

Cellular responses to DNA damage are important determinants of both cancer development and cancer outcome following chemotherapy and radiation therapy. Dysregulation of the DNA damage response (DDR) is associated with predisposition to cancer development and can result in resistance of tumors to chemotherapy [30]. SMARCA5, a chromatin-remodeling enzyme, was required for DNA-templated events including transcription, DNA replication, and DNA repair [18]. Loss of function of the SMARCA5 can result in neurodevelopmental disorder [31] and Williams syndrome [32]. SMARCA5 is a key regulator of chromatin structure and plays pivotal roles in multiple repair pathways that maintain genome stability and prevent cancer [33–35]. SMARCA5 co-localized with CTCF and H2A.Z to control nucleosome repeat length [18]. SMARCA5 is also an upstream sensor protein, which plays key roles in DNA damage signaling cascade [26, 36, 37]. For instance SMARCA5 was found to regulate the ubiquitin response by promoting RNF168 accumulation at DSBs, which subsequently facilitates efficient ubiquitin conjugation and BRCA1 assembly [23]. Hence, targeting of SMARCA5 has recently shown promise as a chemotherapeutic strategy due to increased genomic instability in cancer. The SMARCA5 inhibitor ED2-AD101 was applied to modulate SMARCA5 activity in ovarian cancer cells [38]. However, this compound lacked specificity and also targeted the chromodomain-helicase-DNA-binding protein 4 (CHD4), resulting in off-target effects. Hence, new biomarkers in cancers associated with SMARCA5 need to be discovered. Whether and how the SMARCA5 is regulated upon DDR remain unclear.

Post-translational modification (PTM) is important for its function in DNA repair and tumor radiosensitivity [39, 40]. Deubiquitination, has more recently come to the forefront of DDR research as an important new angle in ubiquitin-mediated regulation of DDR and emerged as key factors in DDR [41]. Importantly, deubiquitinases are attractive small-molecule drug targets due to their well-defined catalytic residues that provide a promising avenue for developing new cancer therapeutics. Here, we found USP3 was frequently upregulated in PCa and correlates with prostate cancer progression. USP3 knockdown inhibit proliferation and survival of prostate cancer cells in vitro and in vivo. Moreover, we carried out a screen for potential USP3’s substrates by mass spectrometry (MS) and identified the USP3 directly interacts with SMARCA5, leading to deubiquitination and stabilization of SMARCA5, which in turn regulates the DNA Damage Response (DDR) in the SMARCA5 dependent manner. Future studies will be needed to investigate the physiological significance of USP3 stabilization of SMARCA5 in vivo.

In addition, we found USP3 is overexpressed in prostate cancer samples and GEO database and showed that high expression of USP3 is related to prostate cancer development. Moreover, a positive correlation between USP3 and SMARCA5 protein levels was observed in the prostate cancer human tissues supporting a role for the activity of the USP3-SMARCA5 axis in human prostate cancer cells. Depletion of USP3 or SMARCA5 promoted PCa cells sensitive to docetaxel and overexpression of USP3 restored the cells resistance to docetaxel treatment in SMARCA5 silenced cells. These results suggested that USP3 may regulate DSB end resection and cellular response to DNA damage in a SMARCA5 dependent manner. USP3 may also regulate DDR through other substrate such as CHK1 [16], BRCA1 [42]. Our team will be interesting to further investigate the molecular mechanisms underlying the overexpression of the USP3-SMARCA5 axis in prostate cancer. Nevertheless, our results reveal that USP3 regulates the stability, ubiquitination and thus the function of SMARCA5, providing a mechanistic link between the deubiquitinase USP3 and the SMARCA5-mediated DDR.

In summary, we demonstrate that deletion of USP3 inhibits PCa cell growth in vitro and in vivo. USP3 directly binds SMARCA5 and removed K63-linked polyubiquitination of SMARCA5 to maintain its stability, which promotes DNA damage repair and chemotherapy resistance. Clinically, USP3 levels are positively correlated with SMARCA5 in prostate tumors with high Gleason. In addition, we provide a proof of-concept study showing that targeting USP3-SMARCA5 axis could be a valuable strategy to treat USP3/SMARCA5-overexpressing chemotherapy-resistant patients and improve drug treatment.

## Supplementary information

Supplementary information

WB-raw data

## Data Availability

The datasets used and/or analyzed during the current study are available from the corresponding author on reasonable request.
