# Supplementary material for: USP3 promotes DNA damage response and chemotherapy resistance through stabilizing and deubiquitinating SMARCA5 in prostate cancer
Source: Cell Death Dis. 2024 Nov 5;15(11):790. doi: 10.1038/s41419-024-07117-3 (PMC11538284; doi:10.1038/s41419-024-07117-3)
Supplement: Supplementary file 1 — Supplementary information [file 41419_2024_7117_MOESM1_ESM.doc]

**Supplementary Data**

**Supplemental Figures, Tables, Materials and Methods, References**

**Supplementary Figures**

**Fig. S1**

**
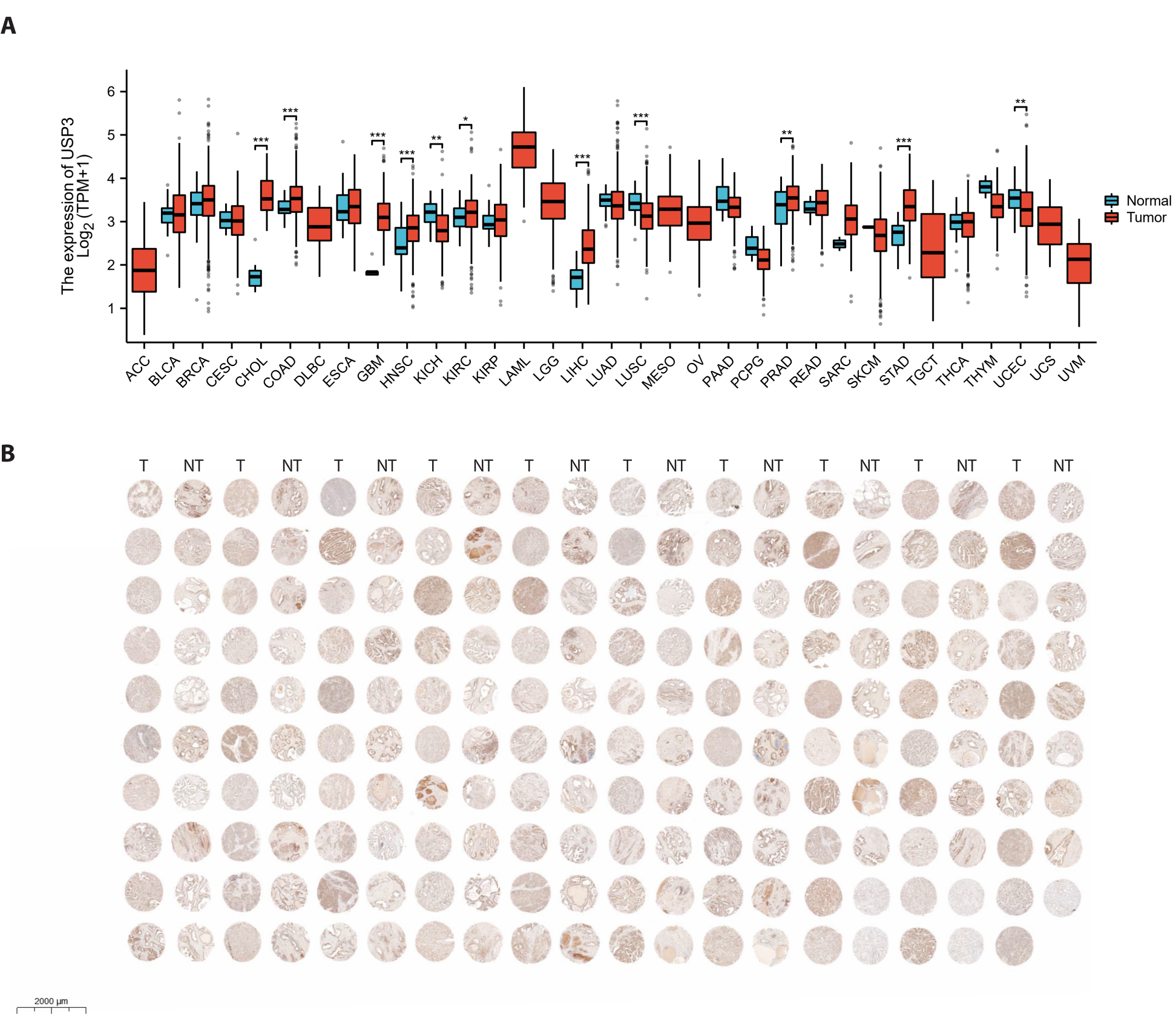
**

**Fig. S1**

(A) The level of USP3 mRNA was analyzed in 33 different types of tumors and normal tissues in the TCGA database (<https://portal.gdc.cancer.gov/>). (B) IHC staining of a PCa tissue microarray including 199 cases with an anti-USP3 antibody. NT: adjacent normal tissue, T: tumor tissue.

**Fig. S2**

**
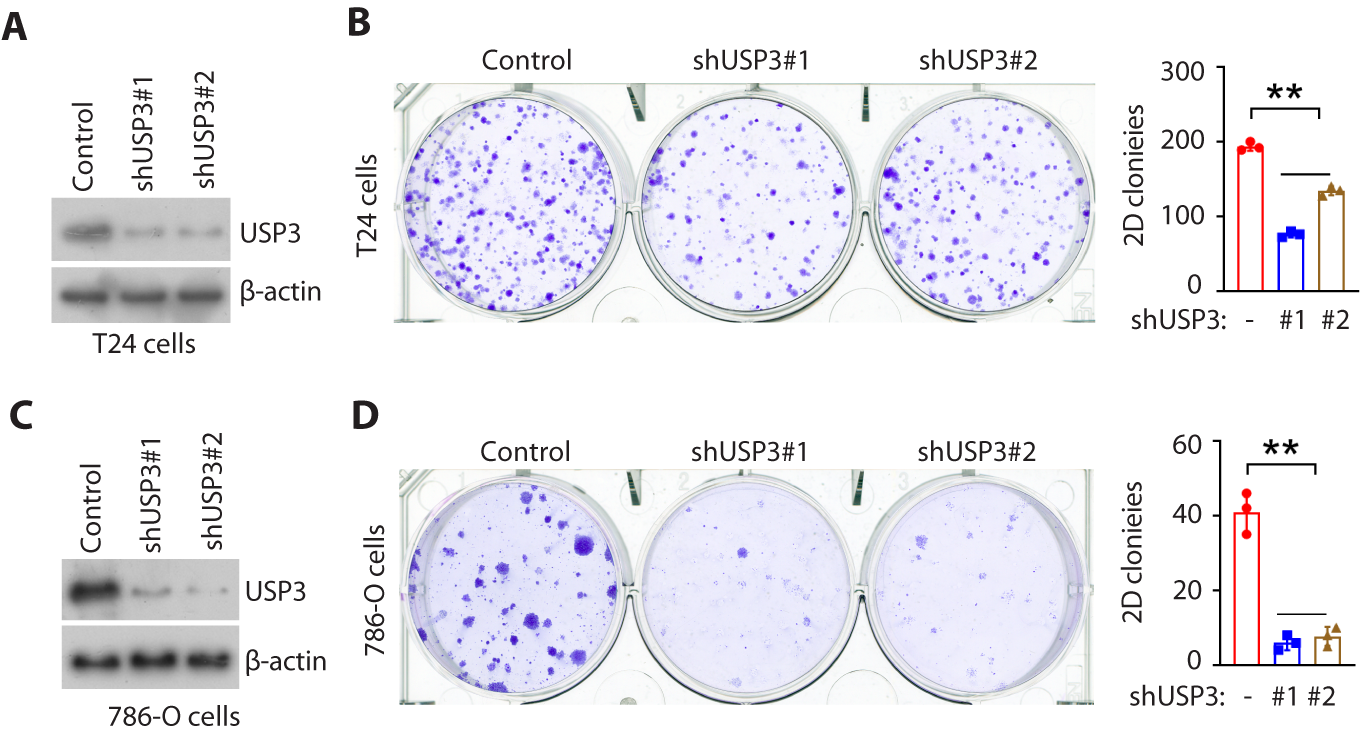
**

**Fig. S2**

(A) USP3 was knocked down in T24 cells by lentivirus control plasmid shUSP3 (#1 and #2), and were detected by Western blot. (B) The cells were generated as in (A) and colony-formation assays were performed (^**^*p* < 0.01). (C) USP3 was knocked down in 786-O cells by lentivirus control plasmid shUSP3 (#1 and #2), and were detected by Western blot. (D) The cells were generated as in (A) and colony-formation assays were performed (^**^*p* < 0.01).

**Fig. S3**

**
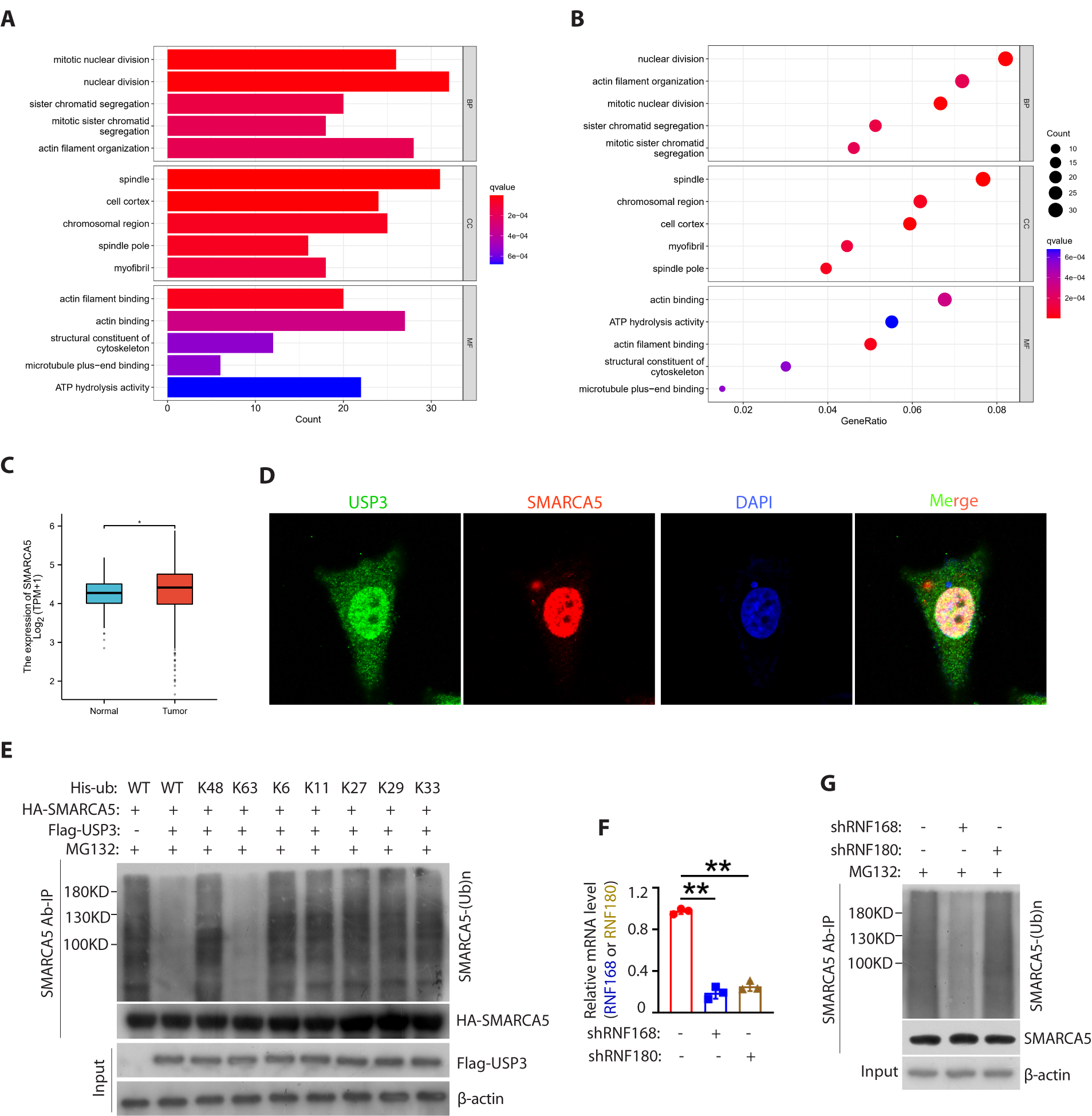
**

**Fig. S3**

(A) (B) The Gene Ontology analysis of USP3 interactome. (C) Expression profile of SMARCA5 in paired primary prostate cancer tissues (n = 496) and matched adjacent non-tumor tissues from XENA database (n = 152). ^*^*p* < 0.05; TCGA. (D) Immunofluorescent staining of USP3 (green) and SMARCA5 (red) in DU145 cells. Nuclear 4', 6-diamidino-2-phenylindole (DAPI; blue). (E) HEK293T cells were transfected with HA-SMARCA5, Flag-USP3, and His-ub (WT, K48, K63, K6, K11, K27, K29 and K33). The analysis was undertaken as described for Fig. 3L. (F) (G) Immunoblotting analysis of the ubiquitination of SMARCA5 in PC3 cells with or without knocking down RNF168 or RNF180. The efficiency of RNF168 or RNF180 knockdown was confirmed by qRT-PCR in PC3 cells. ^**^*p* < 0.01.

**Fig. S4**

**
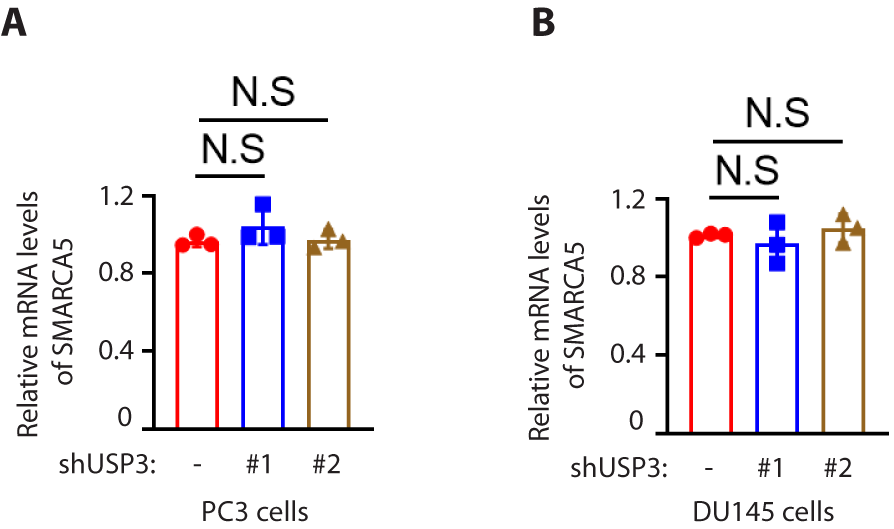
**

**Fig. S4**

(A) (B) USP3 was knocked down in PC3 and DU145 cells by lentivirus control plasmid or shUSP3 (#1 and #2), and mRNA levels of SMARCA5 were detected by qPCR. N.S means no significance.

**Fig. S5**

**
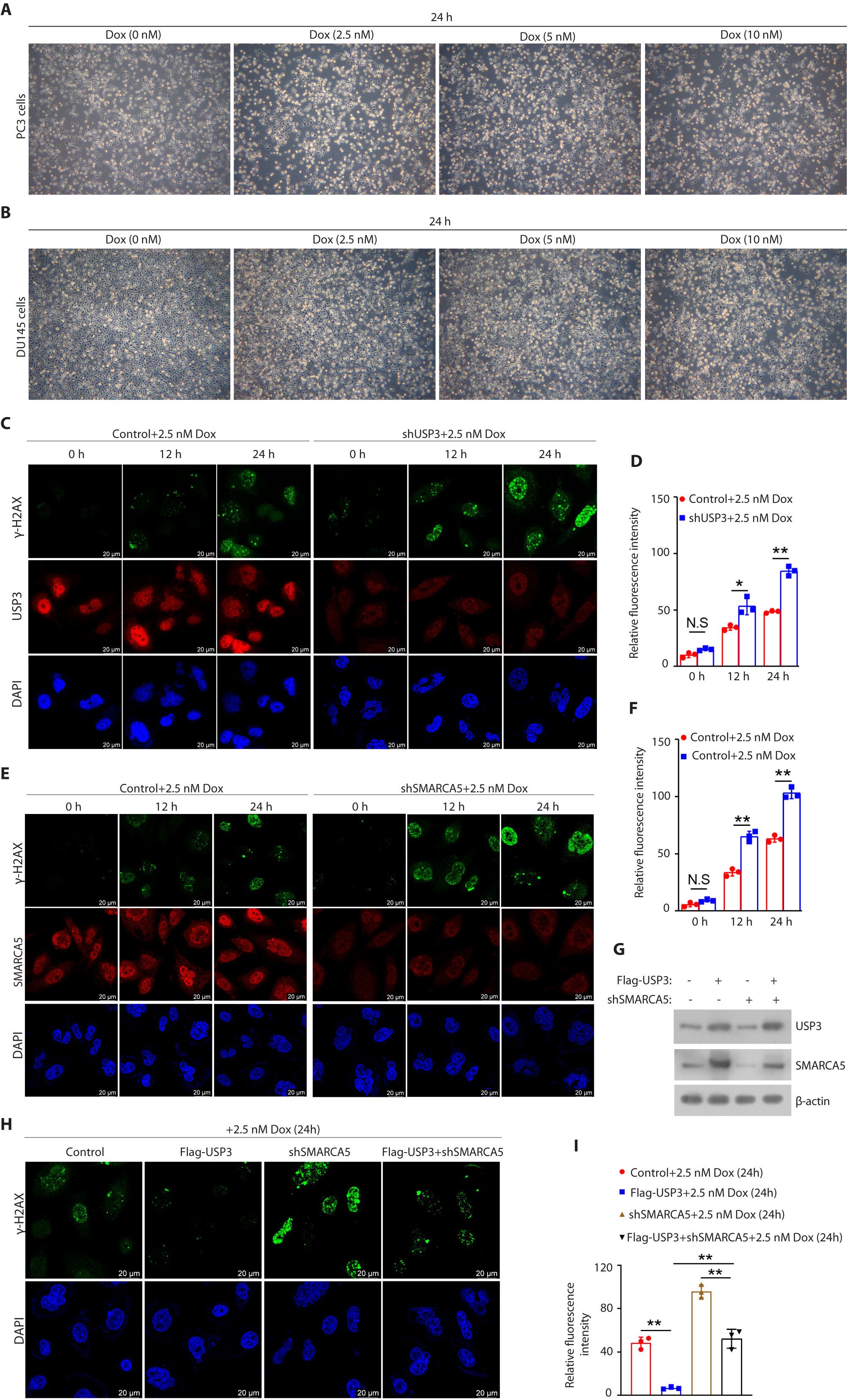
**

**Fig. S5**

(A) Different dose docetaxel treated the PC3 cells for 24h. (B) Different dose docetaxel treated the DU145 cells for 24h. (C) (D) The DU145 cells with or without USP3 silencing were treated with 2.5 nM docetaxel (Dox) at the indicated time points before fixing and processed for γ-H2AX immunofluorescence. N.S means no significance. ^**^*p* < 0.01. (E) (F) The DU145 cells with or without SMARCA5 silencing were treated with 2.5 nM docetaxel (Dox) at the indicated time points before fixing and processed for γ-H2AX immunofluorescence. N.S means no significance. ^**^*p* < 0.01. (G) IB (Immunoblot) analysis of WCLs (whole-cell lysates) derived from SMARCA5 knockdown PC3 cells rescued with Control and Flag-USP3, which were treated with 2.5 nM docetaxel at indicated time points before harvesting. (H) (I) Flag-USP3 rescued SMARCA5 knockdown-mediated high levels of γ-H2AX post-docetaxel treatment at the indicated time points. The DU145 Cells as described in Fig. S5G were fixing and processed for γ-H2AX immunofluorescence. ^**^*p* < 0.01.

**Fig. S6**

**
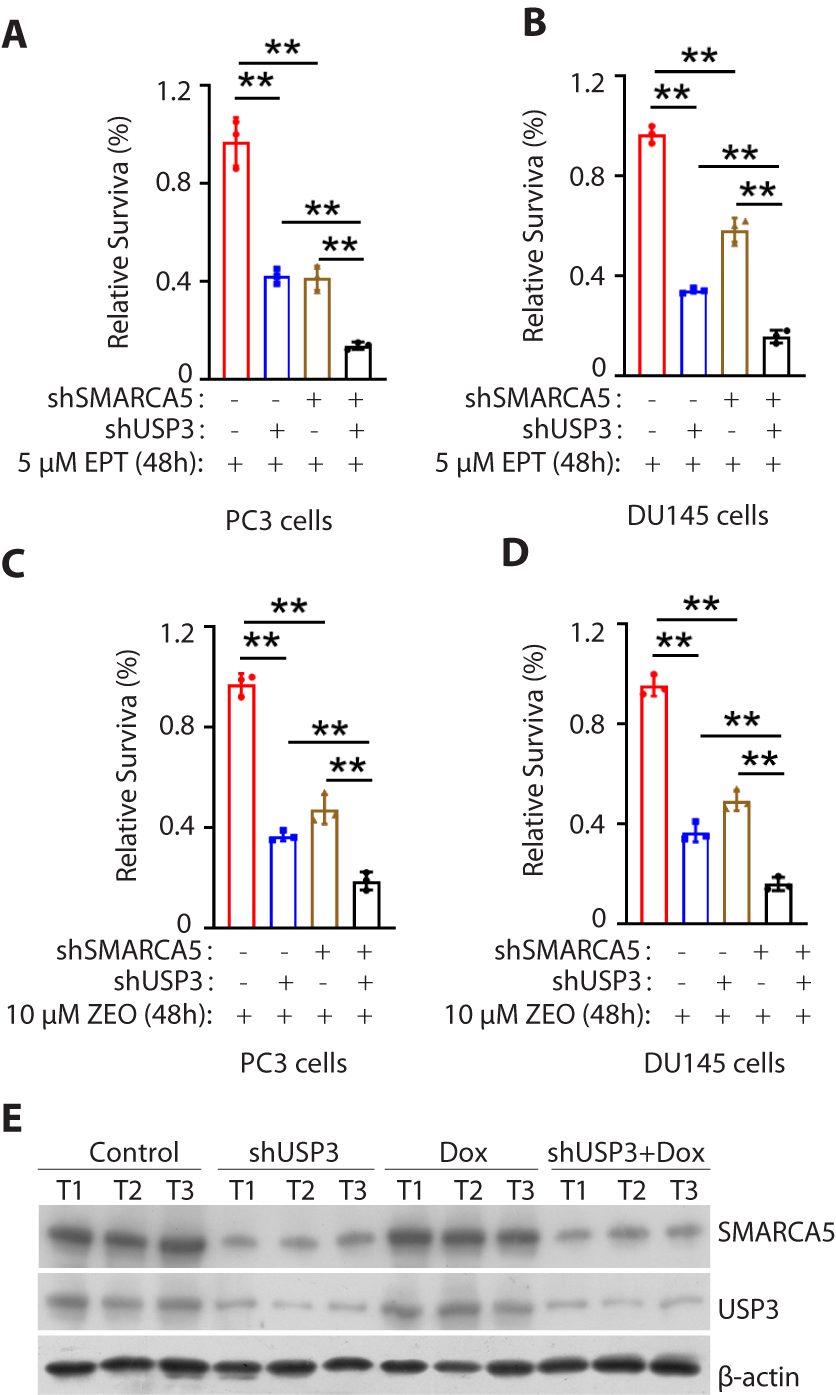
**

**Fig. S6**

(A) (B) The PC3 cells and DU145 cells stably expressing USP3 shRNA were transfected with or without SMARCA5 shRNA and surviving cell percentage was counted after treated 5 μM etoposide (EPT) with for 2 days (mean ± SD, n = 3). ^**^*p* < 0.01. (C) (D) The PC3 cells and DU145 cells stably expressing USP3 shRNA were transfected with or without SMARCA5 shRNA and surviving cell percentage was counted after treated 10 μM zeocin (ZEO) with for 2 days (mean ± SD, n = 3). ^**^*p* < 0.01. (E) **C** The tumor tissues from the indicated nude mice (each group n = 3) were used to detect SMARCA5 and USP3 protein expression.

**Supplementary Tables**

**Table S1** **Clinic-pathological variables and USP3 expression in prostate cancer patients assessed by IHC from the platform.**

Variables All USP3 *P*

n=99 n=30 (low) n=69 (High) value^#^

Age

≤ 65 7 16 0.987

＞ 65 23 53

Tumor stage

T1-T3a 19 26 0.019^*^

T3b-T4 11 43

Stage

I-II 13 14 0.018^*^

III-IV 17 55

Gleason grade

≤ 7 16 18 0.008^**^

> 7 14 51

Lymph node metastasis

No 30 66 0.551

Yes 0 3

^#^*P* value was analyzed by chi-sequared test; ^*^ indicates *P* < 0.05, ^**^*P* < 0.01 with statistical significance.

**Tables S2.** **The candidate ubiquitin-proteins interacted with USP3 identified by Mass Spectrometry.**

| Protein Names | Gene Names | #Peptides | Unique Peptides | Mol. Weight [kDa] |
| --- | --- | --- | --- | --- |
| Ubiquitin carboxyl-terminal hydrolase 3 | USP3 | 48 | 45 | 58.897 |
| Titin | | TTN | | --- | | 13 | 5 | 38.15922 |
| POTE ankyrin domain family member E | POTEE | 11 | 2 | 121.363 |
| Probable rRNA-processing protein EBP2 | EBNA1BP2 | 11 | 10 | 34.852 |
| SWI/SNF-related matrix-associated actin-dependent regulator of chromatin subfamily A member 5 | SMARCA5 | 10 | 7 | 121.905 |

**Table S3** **Clinic-pathological variables and SMARCA5 expression in prostate cancer patients assessed by IHC from the platform.**

Variables All SMARCA5 *P*

n=99 n=29 (low) n=70 (High) value^#^

Age

< 65 10 13 0.088

≥ 65 19 57

Tumor stage

T1-T3a 22 23 < 0.0001^****^

T3b-T4 7 47

Stage

I-II 17 10 < 0.0001^****^

III-IV 12 60

Gleason grade

≤ 7 18 16 0.004^***^

> 7 11 54

Lymph node metastasis

No 29 67 0.553

Yes 0 3

^#^*P* value was analyzed by chi-sequared test; ^*^ indicates *P* < 0.05, ^**^*P* < 0.01 with statistical significance.

**Supplementary Materials and Methods**

**Antibodies and reagents**

Anti-USP3 antibody (Cat No. 12490-1-AP), anti-SMARCA5 antibody (Cat No. 13066-1-AP), anti-β-actin antibody (Cat No. 81115-1-RR) and anti-HA antibody (Cat No. HRP-81290) were purchased from Proteintech (Wuhan, China). Anti-Flag antibody (Cat No. M30971-2) was purchased from BOSTER (Wuhan, China). Anti-SMARCA5 antibody (sc-365727), anti-γ-H2AX antibody (sc-517336) and anti-Ki67 antibody (sc-23900) were purchased from [Santa Cruz](https://cn.bing.com/dict/search?q=Santa Cruz&FORM=BDVSP2&qpvt=santa+cruz)(Dallas, USA). Goat anti-mouse IgG secondary antibody (#7076) and goat anti-rabbit IgG secondary antibody (#7074) were purchased from Cell Signaling Technology (Danvers, USA). Alexa Fluor 488-labeled Goat Anti-Mouse IgG (H+L) and Cy3-labeled Goat Anti-Mouse IgG (H+L) were purchased from Beyotime Biotechnology (Shanghai, China). All were used according to the manufacturers’ recommendations.

**Plasmids and cloning**

Flag-USP3 (P54490), Flag-USP3 (159-520aa) (G26994), Flag-USP3 (1-158aa) (G26993), HA-SMARCA5 (P54716) and HA-SMARCA5 (487-638aa) (G27712) expression plasmid was generated by subcloning the into pCMV-3 vector and were obtained from MiaoLingBio, China (http://www.miaolingbio.com/). His-Ubiquitin-K6 (P63701), His-Ubiquitin-K11 (P63702), His-Ubiquitin-K27 (P63703), His-Ubiquitin-K29 (P63704), His-Ubiquitin-K33 (P63891), His-Ubiquitin-K48 (P63705) and His-Ubiquitin-K63 (P63706) were obtained from MiaoLingBio, China (http://www.miaolingbio.com/). USP3 mutant was generated by using the QuikChange II Site-Directed Mutagenesis Kit. Primers used for cloning are available upon request.

**shRNA lentiviral vector packaging and transduction**

shRNA lentiviral vector packaging and transduction were described as previously[^1^](#_ENREF_1). lentiviral vectors shRNA-USP3, shRNA-RNF168, shRNA-RNF180 and shRNA-GFP pLKO1 (control vector) were purchased from QEgene (Shanghai, China). Lentiviral vector encoding shRNA was packaged in 293T cells by calcium phosphate transfection. The supernatants that contained lentiviral particles was collected 48h after transfection. The indicated PCa cells were then transduced with the supernatant in the presence of polybrene (8 μg/mL) for 24 h before replacement with fresh growth media. Cells were analyzed at 48 or 72 h post transduction.

**Quantitative real-time PCR (qRT-PCR)**

qRT-PCR was described as previously[^1^](#_ENREF_1). Primers for qPCR analysis of human gene transcripts were:

USP3:

Forward Primer: 5’-CATCACCCGCACTTCAG-3’

Reverse Primer: 5’-TCAATCAGGATAGGCCAGA-3’

SMARCA5:

Forward Primer : 5’-TGCAGGTTGGATGGTCAGACAC-3’

Reverse Primer : 5’-GTCGCAAGATTGATGCCAAGACC-3’

RNF168:

Forward Primer: 5’-GGATCTGCATGGAAATCCTCG-3’

 Reverse Primer: 5’-ACTGGAAGCACGGTTTACACA-3’

RNF180:

Forward Primer: 5’-TCTGACTTTCCTGATGGACCTG-3’

 Reverse Primer: 5’-CCTGAGTATTTACCCTGCTTCTGT-3’

β-actin：

Forward Primer: 5’-TCTCCCAAGTCCACACAGG-3’

Reverse Primer: 5’-GGCACGAAGGCTCATCA-3’

Western blotting

Proteins were prepared as previously described[^2^](#_ENREF_2). Briefly, protein extracts were separated by gradient SDS-PAGE gel and then electroblotted onto a PVDF membrane (Cytiva, catalog number: 10600021). The membranes were incubated with the indicated primary antibodies at 1: 1000 at 4°C overnight, respectively, followed by incubation with corresponding secondary antibodies at 1: 10000 at room temperature for 1 h.

Cell proliferation and clony formation assays

Cell proliferation and clony formation were described as previously[^3^](#_ENREF_3).

**Wound healing**

Wound healing assays were performed as described previously[^4^](#_ENREF_4)

**Edu assay**

Edu assays were performed as described previously[^4^](#_ENREF_4).

**Protein immunoprecipitation (IP) and Liquid Chromatography-MS Analysis**

Co-IP or IP was described as previously[^4^](#_ENREF_4). In brief, cell lysates were captured on Flag-M2 beads or protein A/G agarose beads (Santa Cruz, USA). The complexes were then separated, and the gels were stained with silver or were detected by Western blotting. For Liquid Chromatography-MS Analysis, immunoprecipitation of USP3 antibodies was performed as described above. The precipitated proteins were eluted 3 times with lysis buffer. The eluted samples were subjected to in-solution trypsin digestion, followed by liquid chromatography-MS analysis and Protein identification was performed as previously described[^4^](#_ENREF_4).

Ubiquitination assay

Ubiquitination assay was described as previously[^4^](#_ENREF_4). In brief, the cells were transfected with indicated plasmids and lysed with the immunoprecipitation buffer. For immunoprecipitation, 2 mg of protein was incubated with indicated antibodies at 4°C overnight before Flag-M2 beads or protein A/G beads were added for 2 h. Beads were washed once with TBS, 1% Triton X-100, 1% SDS, twice with 0.5 M LiCl, TBS buffer and again in PBS 1% Triton X-100 containing buffer. Proteins were loaded onto 8% SDS-PAGE gels and immunoblotted with the indicated antibodies.

**Immunostaining**

The indicated PCa cells were plated on cover glasses then incubated with each reagent for the appropriate amount of time. They were then fixed in 4% paraformaldehyde for 10min. And then, coverslips were washed twice with PBS. Following this, cells were blocked with 5% goat serum for 30 min and then washed one time with PBS. And incubated with primary antibodies. Next day, after washing with PBS for three times, secondary antibodies diluted in 3% BSA in PBS for 1 h at room temperature. DAPI was used to locate cell nuclei. The cells staining in 3 randomly selected fields was photographed by fluorescence microscopy (Olympus, Japan).

Supplemental References

1. Xu S, Fan L, Jeon HY, Zhang F, Cui X, Mickle MB*, et al.* p300-Mediated Acetylation of Histone Demethylase JMJD1A Prevents Its Degradation by Ubiquitin Ligase STUB1 and Enhances Its Activity in Prostate Cancer. *Cancer research* 2020, **80**(15)**:** 3074-3087.

2. Xu SH, Huang JZ, Xu ML, Yu G, Yin XF, Chen D*, et al.* ACK1 promotes gastric cancer epithelial-mesenchymal transition and metastasis through AKT-POU2F1-ECD signalling. *The Journal of pathology* 2015, **236**(2)**:** 175-185.

3. Tang DE, Dai Y, He JX, Lin LW, Leng QX, Geng XY*, et al.* Targeting the KDM4B-AR-c-Myc axis promotes sensitivity to androgen receptor-targeted therapy in advanced prostate cancer. *The Journal of pathology* 2020, **252**(2)**:** 101-113.

4. Xiong S, Li S, Li Z, Song Y, Yang L, Yang H*, et al.* A noncanonical E3 ubiquitin ligase RNF41-mediated MYO1C stability promotes prostate cancer metastasis by inducing actin remodeling. *Oncogene* 2024 Aug 7. doi: 10.1038/s41388-024-03120-2. Epub ahead of print. PMID: 39112516.
