# Supplementary material for: USP3 promotes DNA damage response and chemotherapy resistance through stabilizing and deubiquitinating SMARCA5 in prostate cancer
Source: Cell Death Dis. 2024 Nov 5;15(11):790. doi: 10.1038/s41419-024-07117-3 (PMC11538284; doi:10.1038/s41419-024-07117-3)
Supplement: Supplementary file 2 — WB-raw data [file 41419_2024_7117_MOESM2_ESM.pdf]

Western Blot, Raw data

Figure 1

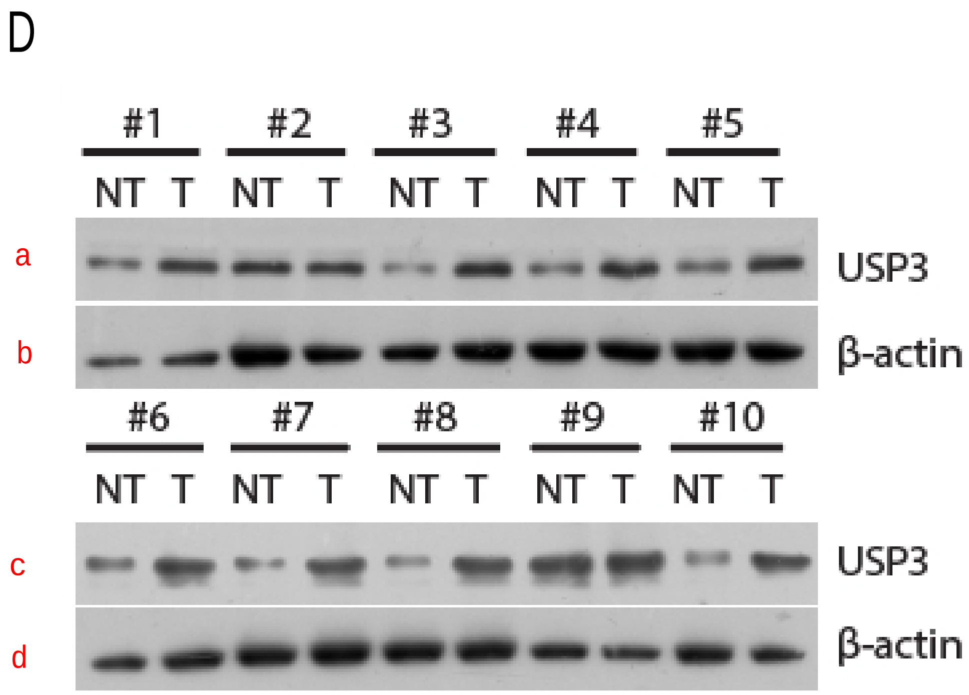

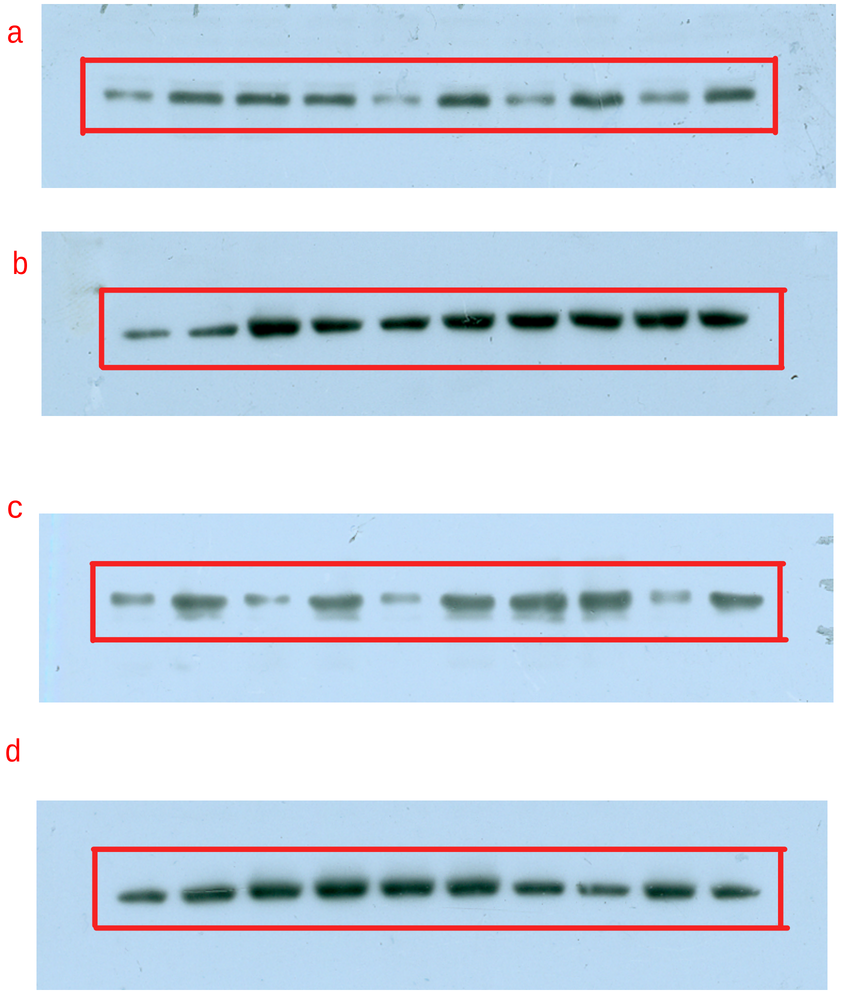

Figure 2

B

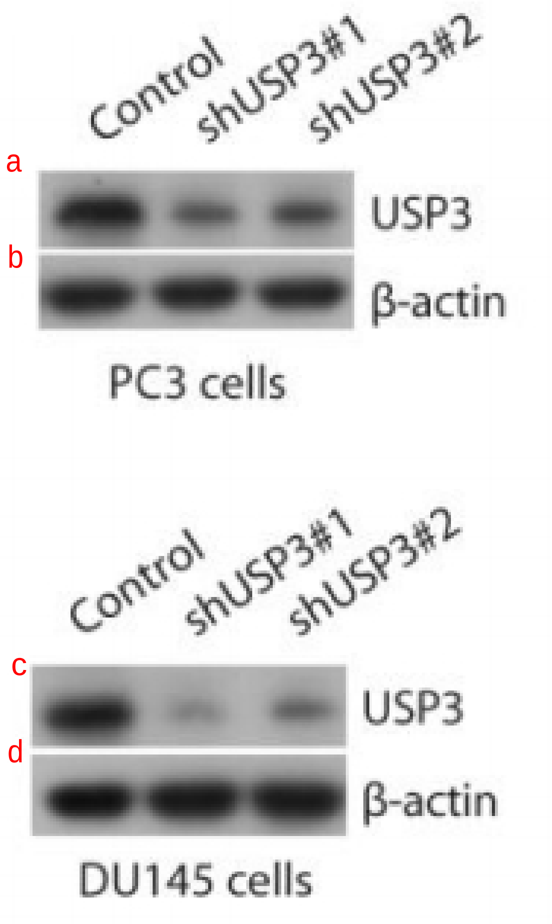
Control
shUSP3#1
shUSP3#2
a
USP3
b
β-actin
PC3 cells
Control
shUSP3#1
shUSP3#2
c
USP3
d
β-actin
DU145 cells

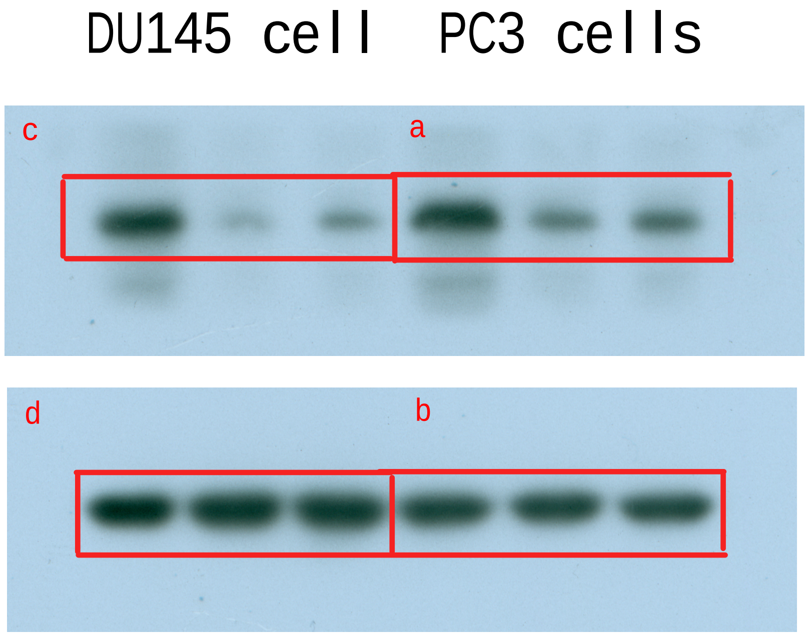
DU145 cell
PC3 cells
c
a
d
b

Figure 3

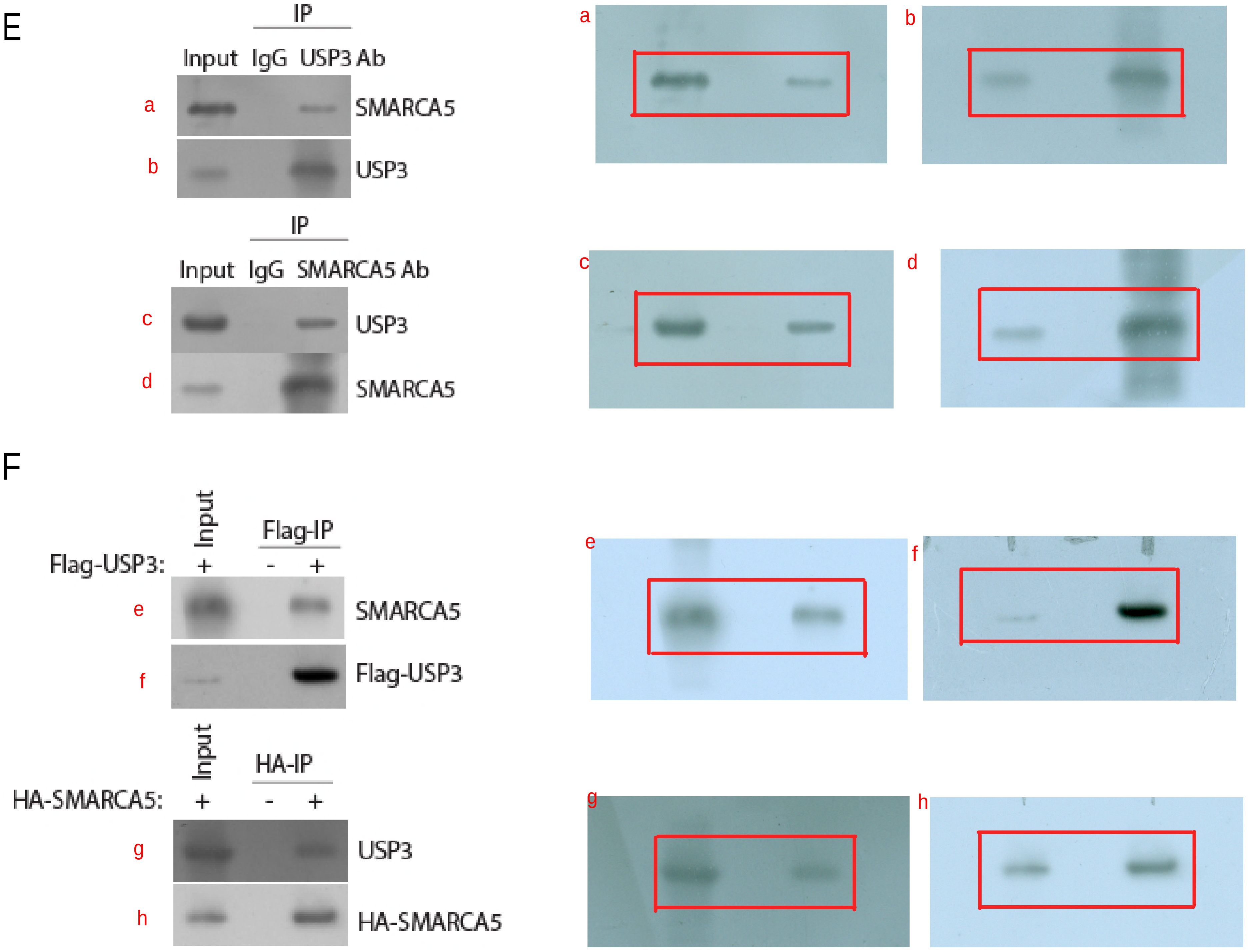
E
IP
Input IgG USP3 Ab
a
SMARCA5
b
USP3
IP
Input IgG SMARCA5 Ab
c
USP3
d
SMARCA5
F
Input
Flag-IP
Flag-USP3: + - +
e
SMARCA5
f
Flag-USP3
Input
HA-IP
HA-SMARCA5: + - +
g
USP3
h
HA-SMARCA5
a
b
c
d
e
f
g
h

Figure 3

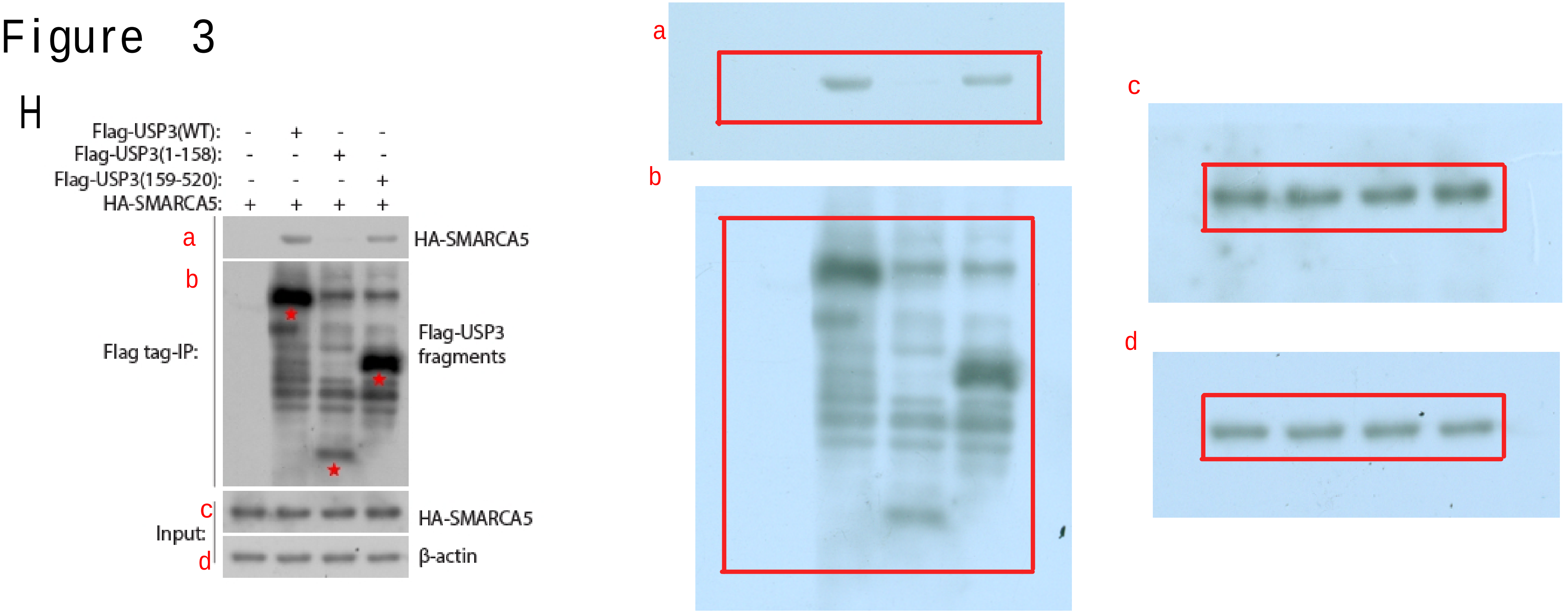

H
Flag-USP3(WT): - + - -
Flag-USP3(1-158): - - + -
Flag-USP3(159-520): - - - +
HA-SMARCA5: + + + +
a
b
Flag tag-IP:
HA-SMARCA5
Flag-USP3 fragments
c
Input:
d
HA-SMARCA5
β-actin
a
b
c
d

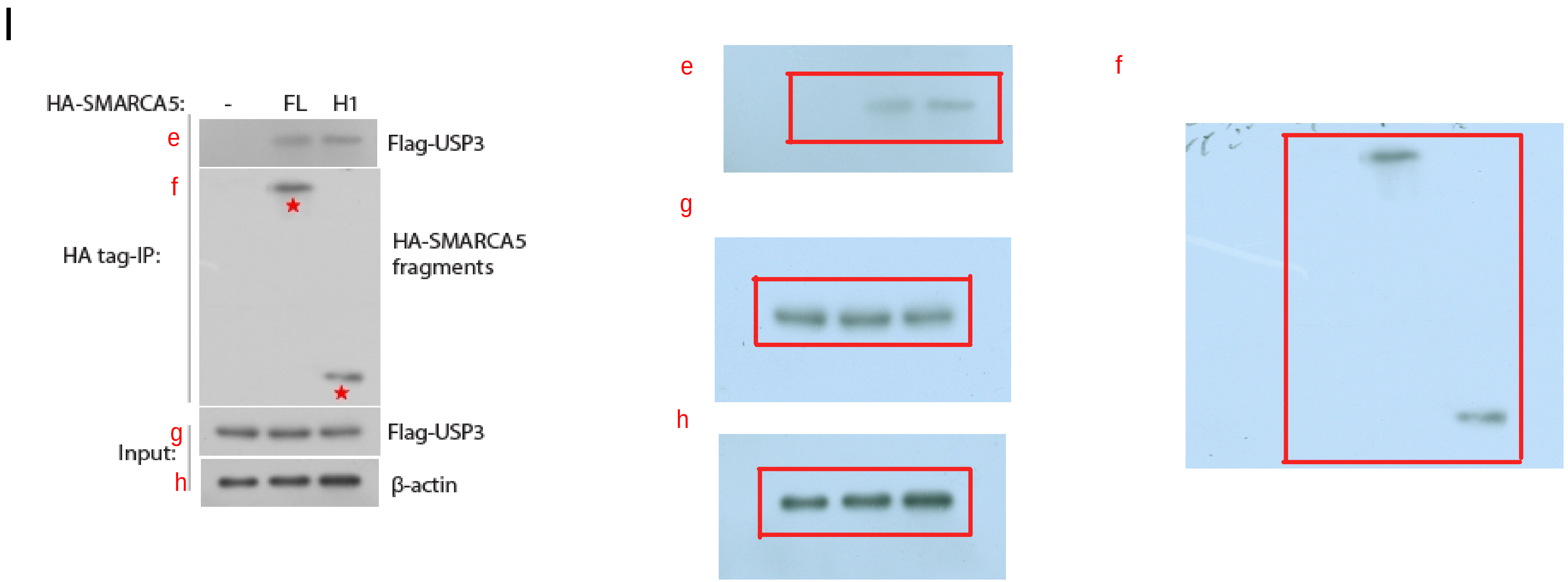

I
HA-SMARCA5: - FL H1
e
f
HA tag-IP:
Flag-USP3
HA-SMARCA5 fragments
g
Input:
h
Flag-USP3
β-actin
e
f
g
h

Figure 3

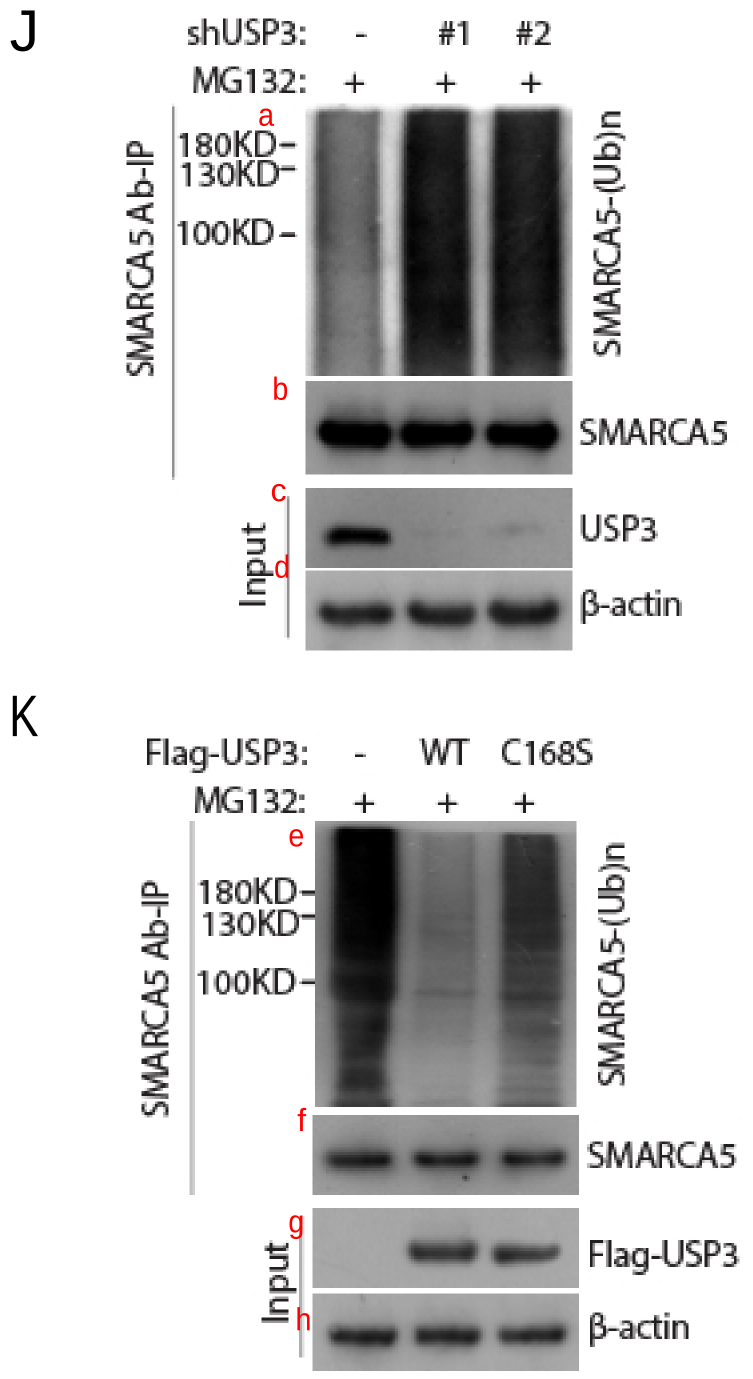
J
shUSP3: - #1 #2
MG132: + + +
a
180KD
130KD
100KD
SMARCA5 Ab-IP
SMARCA5-(Ub)n
b
SMARCA5
c
USP3
Input
d
β-actin
K
Flag-USP3: - WT C168S
MG132: + + +
e
180KD
130KD
100KD
SMARCA5 Ab-IP
SMARCA5-(Ub)n
f
SMARCA5
g
Flag-USP3
Input
h
β-actin

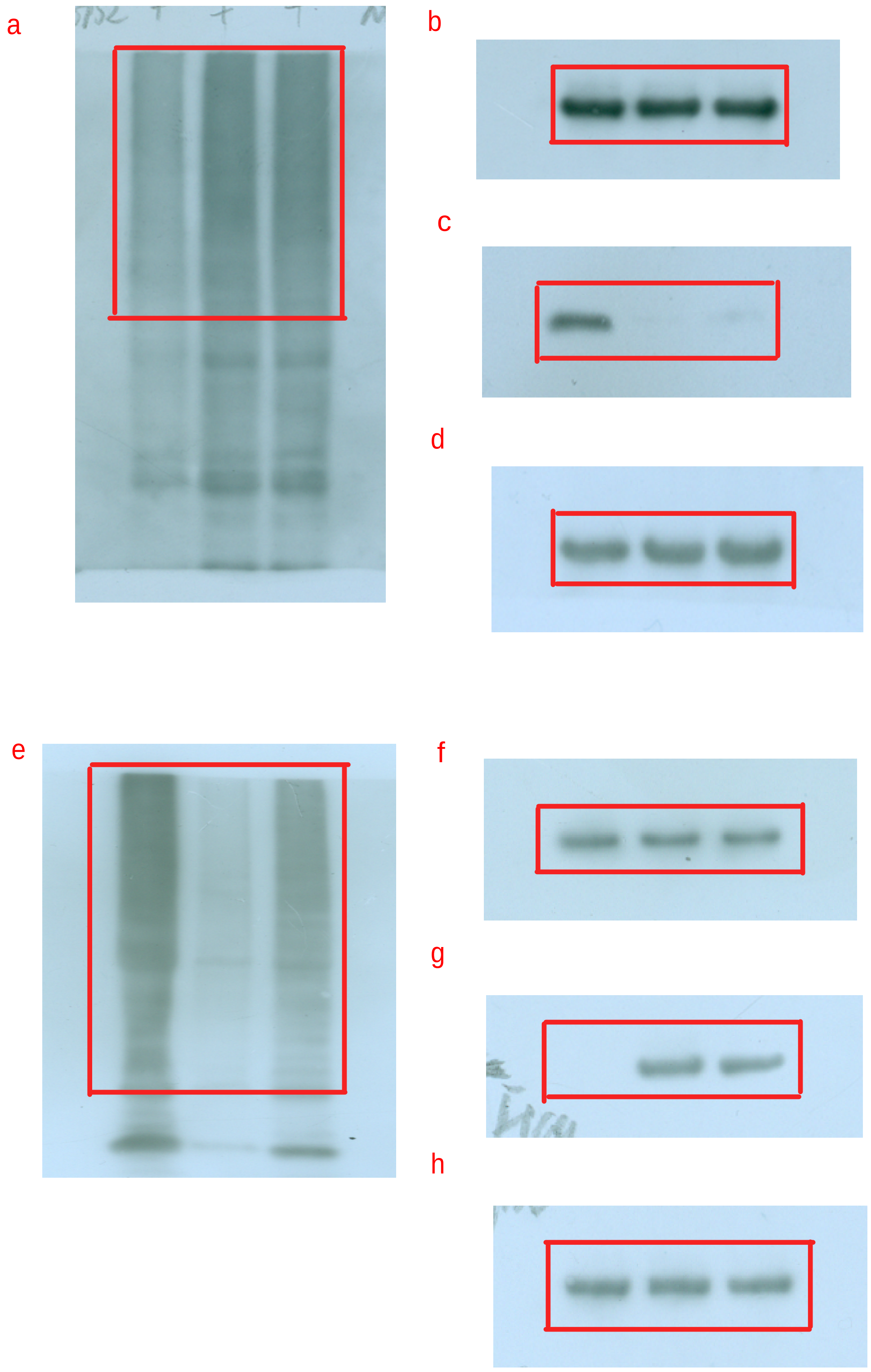
a
b
c
d
e
f
g
h

Figure 3

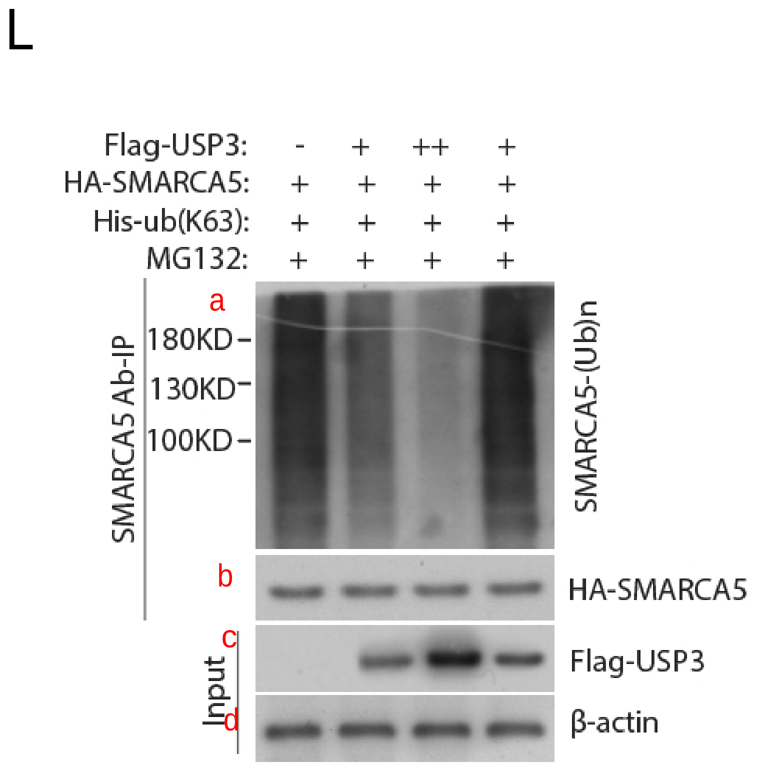

L
Flag-USP3: - + ++ +
HA-SMARCA5: + + + +
His-ub(K63): + + + +
MG132: + + + +
SMARCA5 Ab-IP
a
180KD
130KD
100KD
SMARCA5-(Ub)n
b
HA-SMARCA5
c
Flag-USP3
Input
d
β-actin

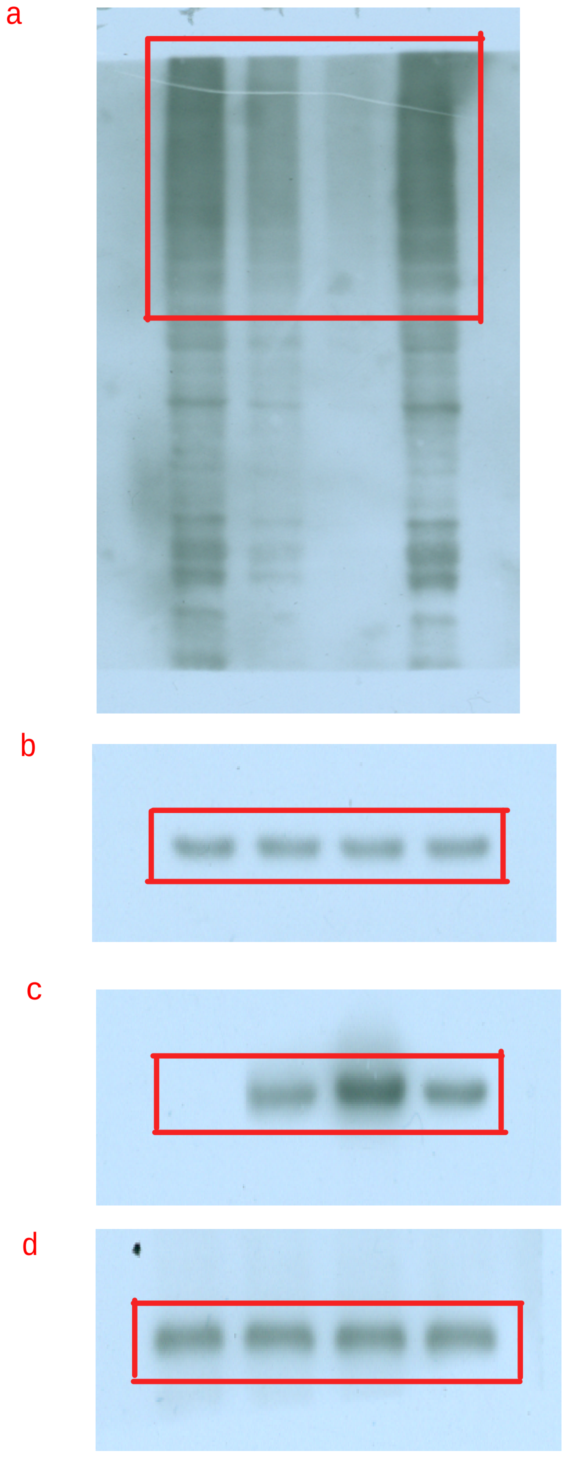

a
b
c
d

Figure 4

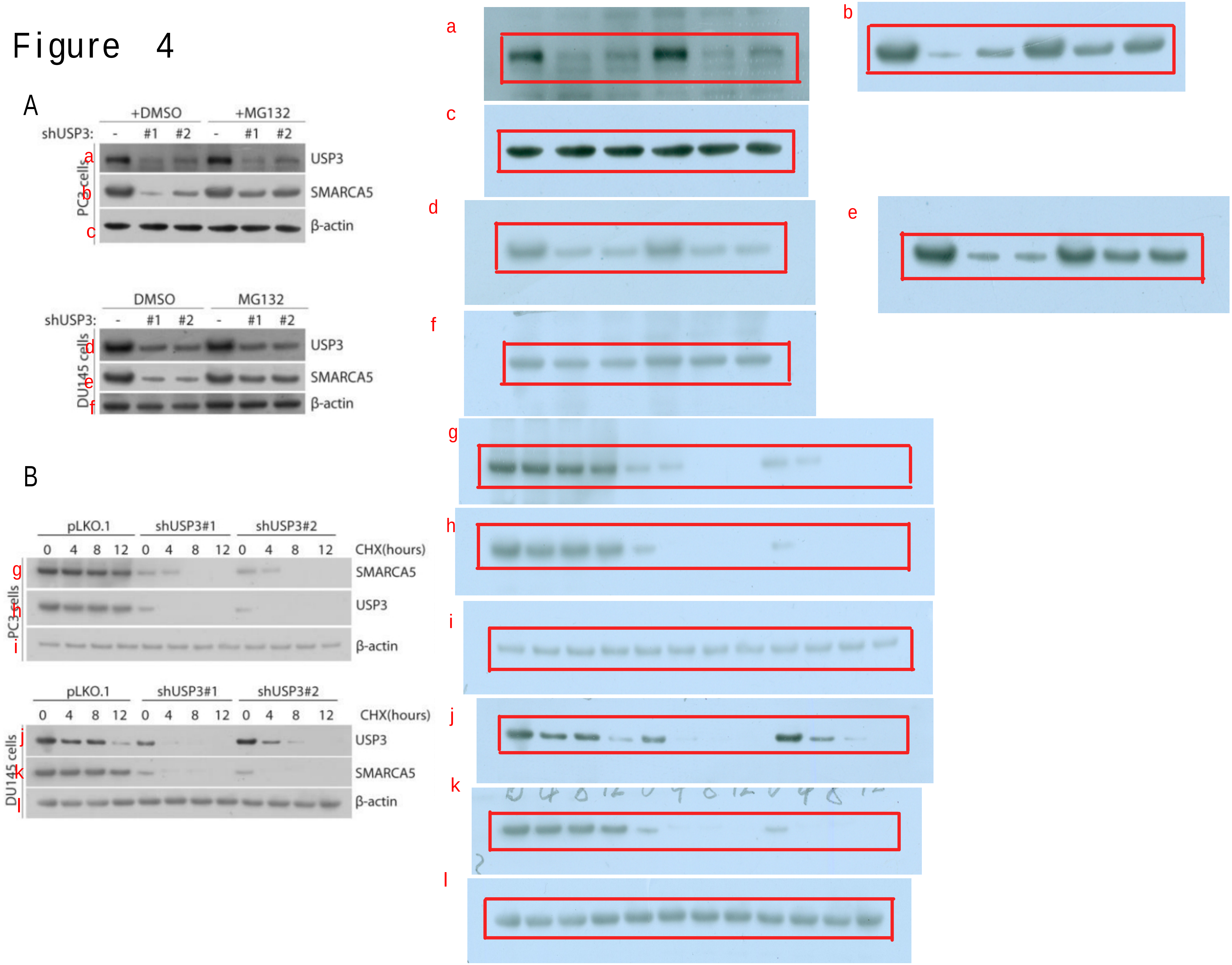
A
+DMSO
+MG132
shUSP3:
-
#1
#2
PC3 cells
USP3
SMARCA5
β-actin
DMSO
MG132
DU145 cells
B
pLKO.1
shUSP3#1
shUSP3#2
0 4 8 12 0 4 8 12 0 4 8 12
CHX(hours)
a
b
c
d
e
f
g
h
i
j
k
l

Figure 4

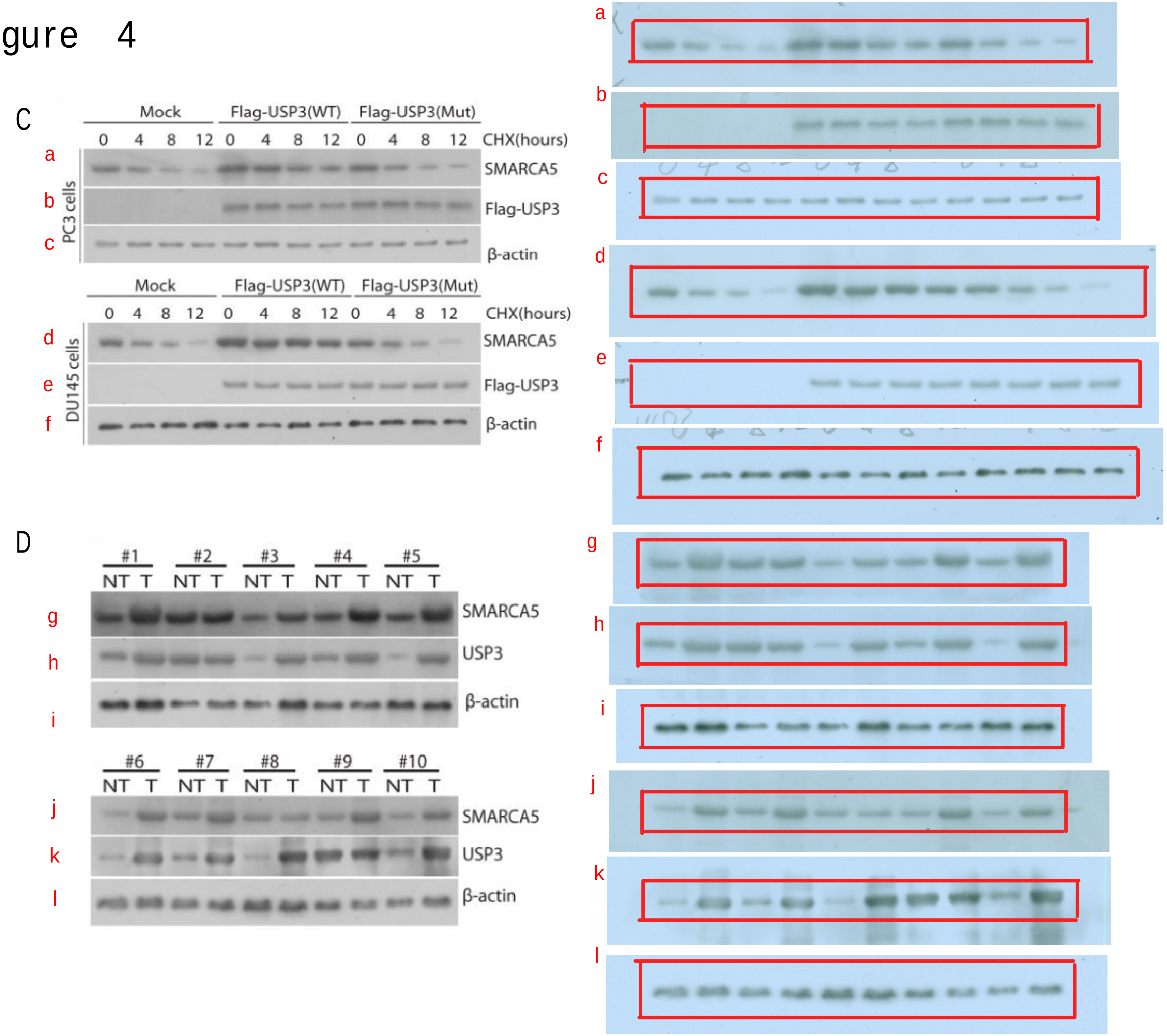
C
Mock
Flag-USP3(WT)
Flag-USP3(Mut)
0 4 8 12 0 4 8 12 0 4 8 12 CHX(hours)
PC3 cells
a
b
c
SMARCA5
Flag-USP3
β-actin
Mock
Flag-USP3(WT)
Flag-USP3(Mut)
0 4 8 12 0 4 8 12 0 4 8 12 CHX(hours)
DU145 cells
d
e
f
SMARCA5
Flag-USP3
β-actin
D
#1 #2 #3 #4 #5
NT T NT T NT T NT T NT T
g
h
i
SMARCA5
USP3
β-actin
#6 #7 #8 #9 #10
NT T NT T NT T NT T NT T
j
k
l
SMARCA5
USP3
β-actin
a
b
c
d
e
f
g
h
i
j
k
l

Figure 6

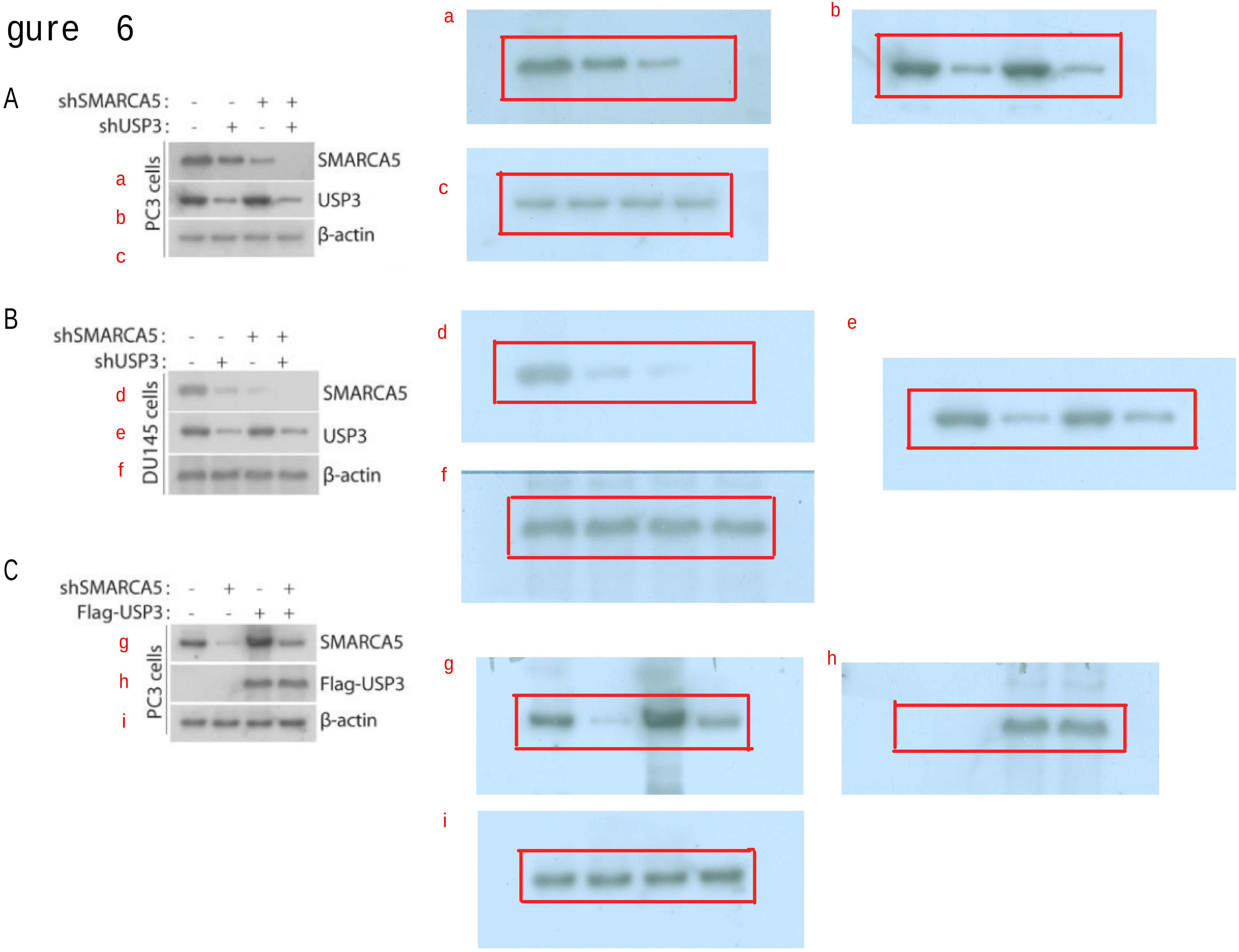
A
shSMARCA5: - - + +
shUSP3: - + - +
PC3 cells
SMARCA5
USP3
β-actin
B
shSMARCA5: - - + +
shUSP3: - + - +
DU145 cells
SMARCA5
USP3
β-actin
C
shSMARCA5: - + - +
Flag-USP3: - - + +
PC3 cells
SMARCA5
Flag-USP3
β-actin
a
b
c
d
e
f
g
h
i

Figure 6

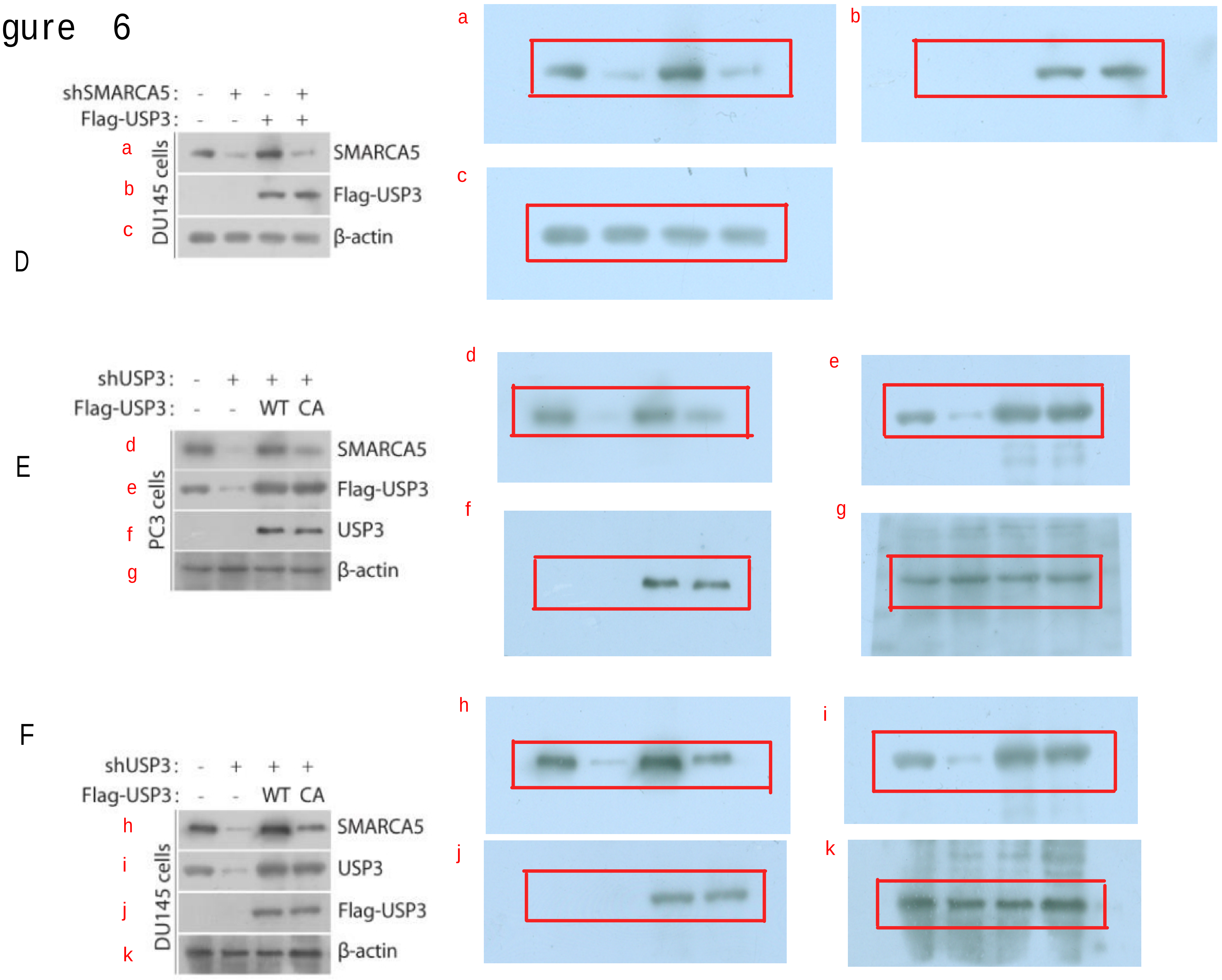
D
shSMARCA5: - + - +
Flag-USP3: - - + +
DU145 cells
SMARCA5
Flag-USP3
β-actin
a
b
c
E
shUSP3: - + + +
Flag-USP3: - - WT CA
PC3 cells
SMARCA5
Flag-USP3
USP3
β-actin
d
e
f
g
F
shUSP3: - + + +
Flag-USP3: - - WT CA
DU145 cells
SMARCA5
USP3
Flag-USP3
β-actin
h
i
j
k

Supplement data Figure

Figure S2 A, C

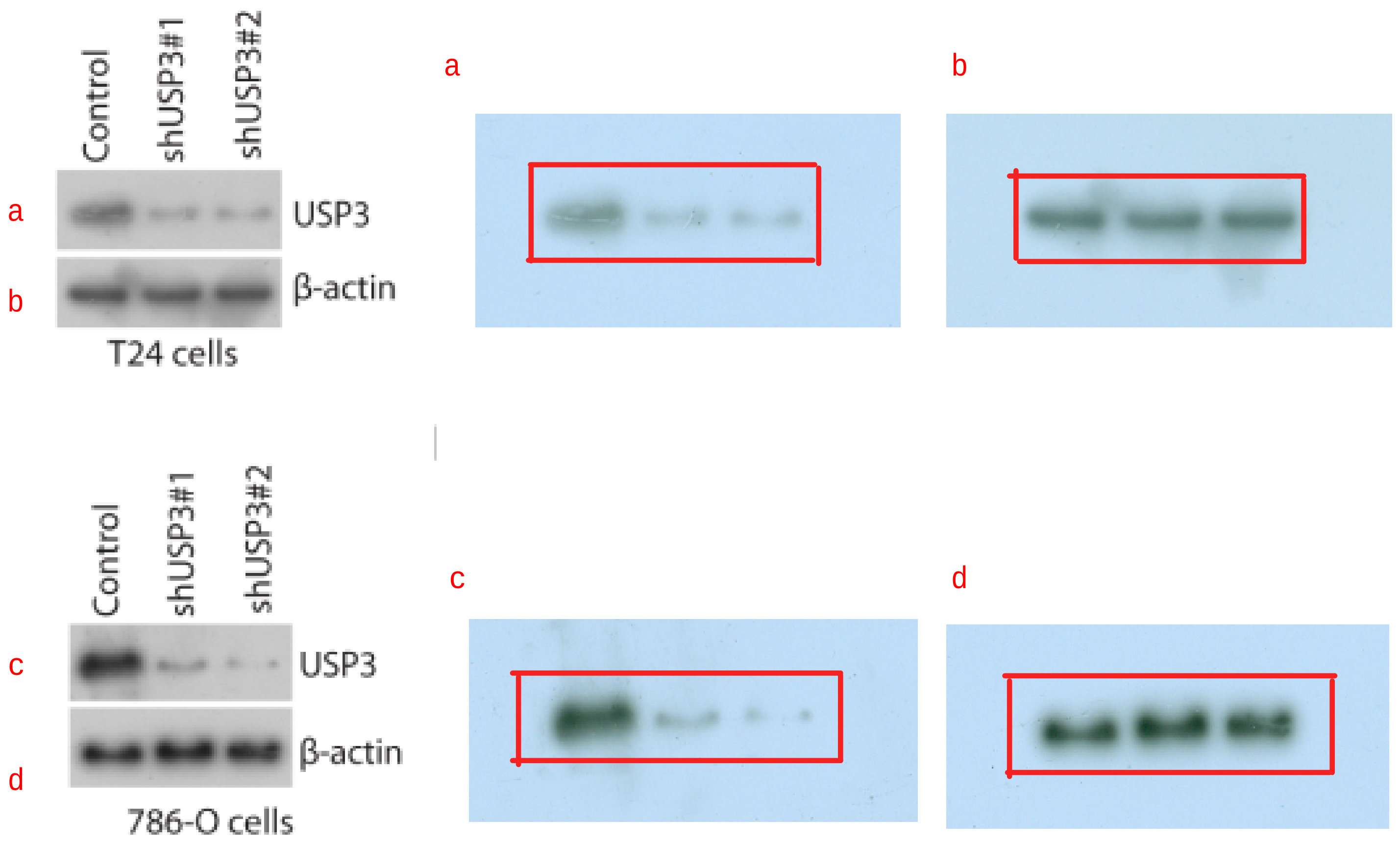

Figure S3 E

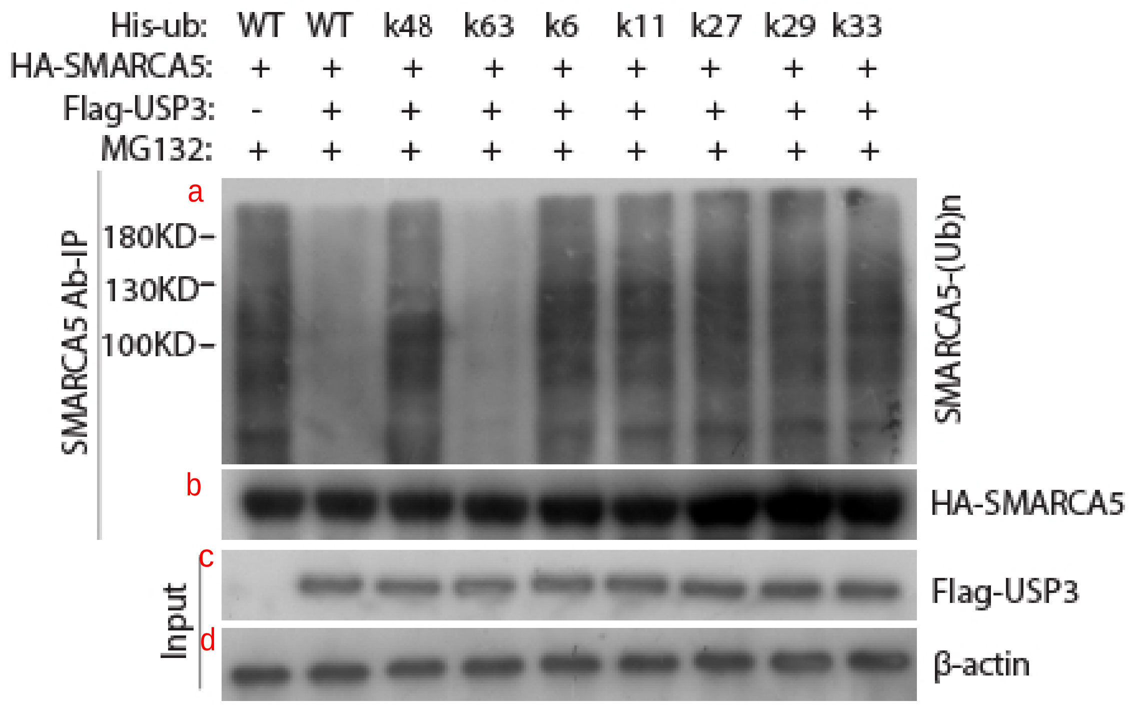
His-ub: WT WT k48 k63 k6 k11 k27 k29 k33
HA-SMARCA5: + + + + + + + + +
Flag-USP3: - + + + + + + + +
MG132: + + + + + + + + +
SMARCA5 Ab-IP
a
180KD
130KD
100KD
SMARCA5-(Ub)n
b
HA-SMARCA5
Input
c
Flag-USP3
d
β-actin

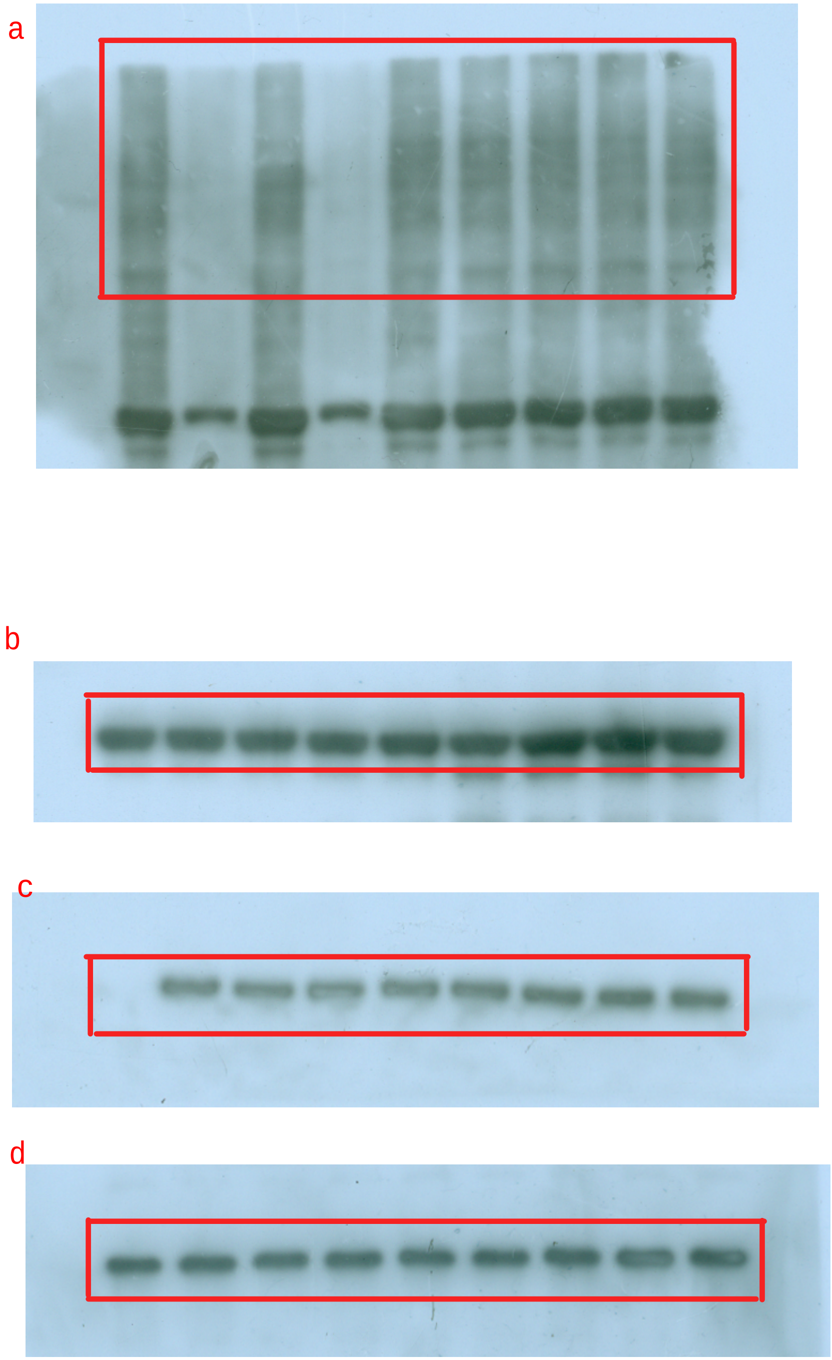
a
b
c
d

Figure S3 G

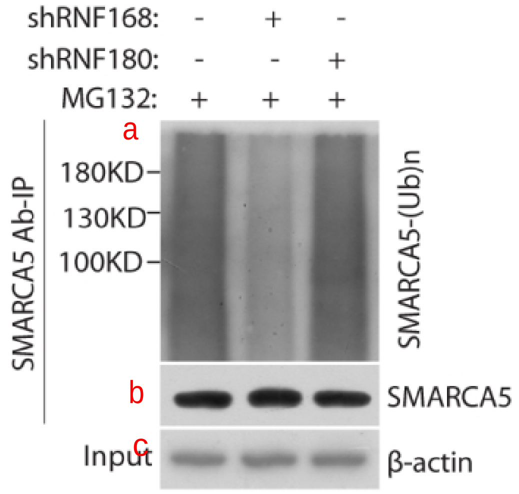

a

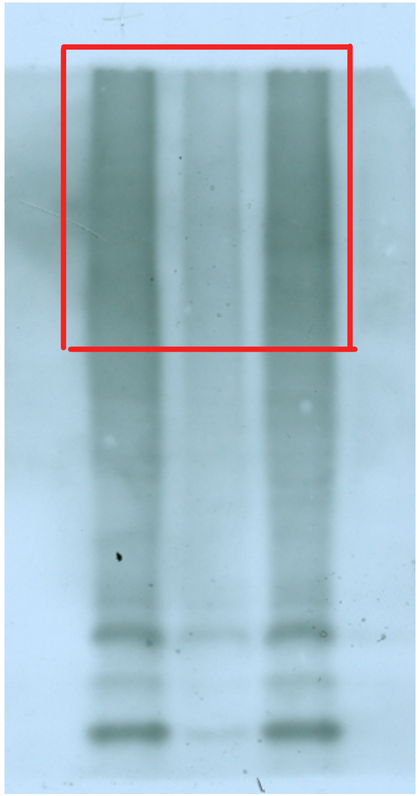

b

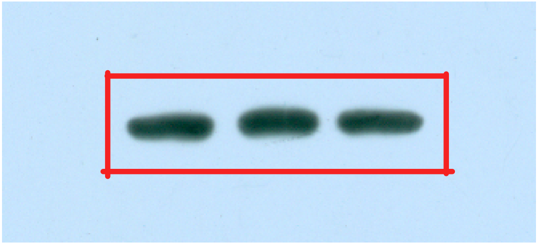

c

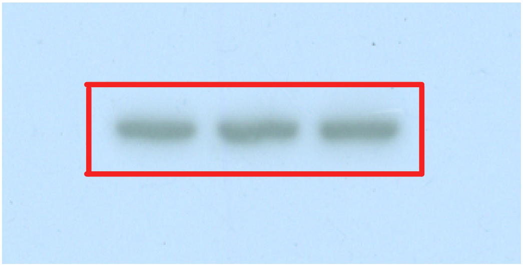

Figure S5 G

C

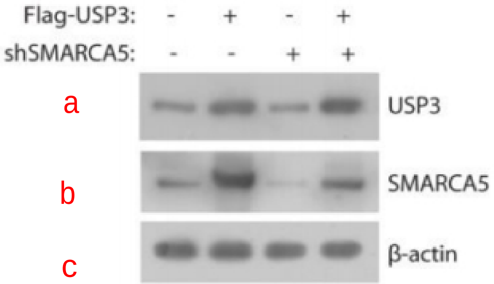

a

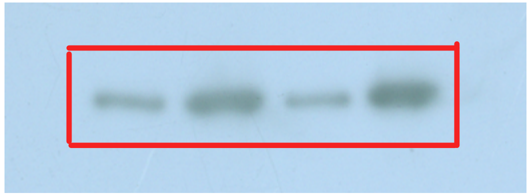

b

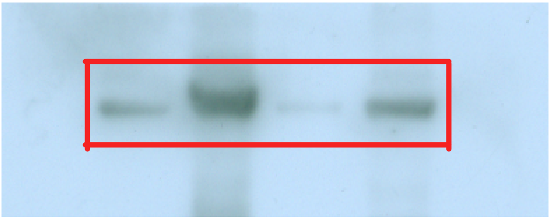

c

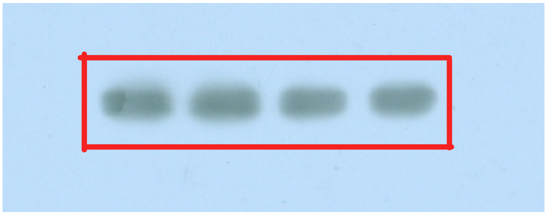

Figure S6 E

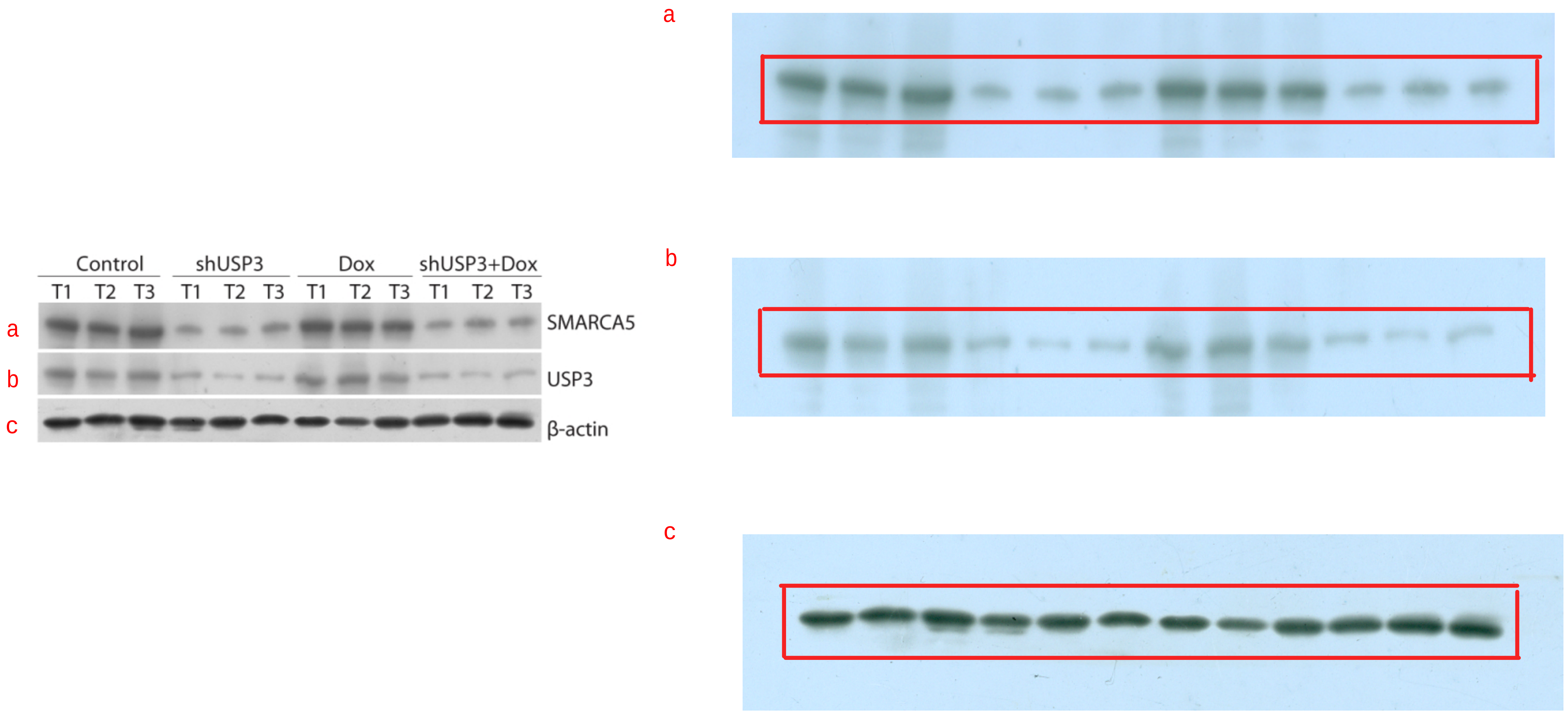
Control
shUSP3
Dox
shUSP3+Dox
T1 T2 T3 T1 T2 T3 T1 T2 T3 T1 T2 T3
a
SMARCA5
b
USP3
c
β-actin
a
b
c
